# Conditional deletion of *Rcan1* predisposes to hypertension-mediated intramural hematoma and subsequent aneurysm and aortic rupture

**DOI:** 10.1038/s41467-018-07071-7

**Published:** 2018-11-15

**Authors:** Silvia Villahoz, Paula Sofía Yunes-Leites, Nerea Méndez-Barbero, Katia Urso, Elena Bonzon-Kulichenko, Sagrario Ortega, J. Francisco Nistal, Jesus Vazquez, Stefan Offermanns, Juan Miguel Redondo, Miguel R. Campanero

**Affiliations:** 1 Gene regulation in cardiovascular remodeling and inflammation group, Centro Nacional de Investigaciones Cardiovasculares (CNIC), Madrid, 28029 Spain; 2 Centro de Investigaciones Biomédicas en RED en Enfermedades Cardiovasculares (CIBERCV), Madrid, Spain; 3 Cardiovascular Proteomics Laboratory, CNIC, Madrid, 28029 Spain; 4 Centro Nacional de Investigaciones Oncológicas (CNIO), Madrid, 28029 Spain; 5 Cardiovascular Surgery, Hospital Universitario Marqués de Valdecilla, IDIVAL, Facultad de Medicina, Universidad de Cantabria, Santander, 39008 Spain; 6 Department of Pharmacology, Max Planck Institute for Heart and Lung Research, Bad Nauheim, 61231 Germany; 7 Department of Cancer Biology, Instituto de Investigaciones Biomedicas Alberto Sols, CSIC-UAM, Madrid, 28029 Spain; 8 Present Address: Vascular Research Lab., IIS-Fundación Jiménez Díaz, Autonomous University of Madrid, Madrid, 28040 Spain; 9 Present Address: ITF Research Pharma, Alcobendas, Madrid, 28108 Spain

## Abstract

Aortic intramural hematoma (IMH) can evolve toward reabsorption, dissection or aneurysm. Hypertension is the most common predisposing factor in IMH and aneurysm patients, and the hypertensive mediator angiotensin-II induces both in mice. We have previously shown that constitutive deletion of *Rcan1* isoforms prevents Angiotensin II-induced aneurysm in mice. Here we generate mice conditionally lacking each isoform or all isoforms in vascular smooth muscle cells, endothelial cells, or ubiquitously, to determine the contribution to aneurysm development of *Rcan1* isoforms in vascular cells. Surprisingly, conditional *Rcan1* deletion in either vascular cell-type induces a hypercontractile phenotype and aortic medial layer disorganization, predisposing to hypertension-mediated aortic rupture, IMH, and aneurysm. These processes are blocked by ROCK inhibition. We find that Rcan1 associates with GSK-3β, whose inhibition decreases myosin activation. Our results identify potential therapeutic targets for intervention in IMH and aneurysm and call for caution when interpreting phenotypes of constitutively and inducibly deficient mice.

## Abstract (web summary)

Constitutive deletion of Rcan1 has been previously shown to prevent Angiotensin II-induced aneurysm in mice. Here the authors show that tissue-specific inducible deletion of Rcan1 in vascular cell types predisposes to hypertension-mediated aortic rupture, intramural hematoma, and aneurysm, due to increased GSK-3b-mediated activation of ROCK and induction of a hypercontractile phenotype.

## Introduction

Pathological vascular wall remodeling, involving structural and functional modifications that destabilize the ordered multilayered organization of the wall, is a central feature of several diseases, including aortic intramural hematoma (IMH) and aortic aneurysm (AA). IMH, a life-threatening acute aortic disease, is a contained hematoma featuring bleeding within the medial layer that weakens the aortic wall. The distinguishing feature of IMH is the absence of the intimal tear or flap formation that characterizes classical aortic dissection. In its early phases, IMH can regress or progress to aortic dissection or rupture, whereas long-duration IMH can progress to aortic aneurysm or pseudoaneurysm^1^. Although the etiology and molecular mechanisms underlying IMH are mostly unknown, it is associated with old age and hypertension^2–4^. Hypertension is also a major risk factor for aortic aneurysm and dissection in humans^5–7^. Indeed, nearly 80% of patients who develop an aortic dissection have hypertension^8,9^. In addition, the hypertensive factor angiotensin II (AngII) induces IMH^10^ and contributes to aneurysm formation in the ascending and the abdominal aorta in animal models^10–13^.

We previously reported AngII-induced expression of regulator of calcineurin 1 (Rcan1) in the aorta^14^. RCAN1, previously known as DSCR1/MCIP1/Calcipressin-1/Adapt78 in mammals, belongs to a family of endogenous regulators of calcineurin activity that also includes RCAN2 and RCAN3^15^. The *RCAN1* gene is expressed as 2 isoforms, *RCAN1-1* and *RCAN1-4*, that differ only in their first exon^15,16^. While *RCAN1-1* seems to be constitutively expressed, *RCAN1-4* transcription is induced de novo by several stimuli that activate the calcineurin-NFAT pathway^14,17–23^. RCAN1 has been implicated in important physiological and pathological processes, including atherosclerosis, aneurysm and neointima formation, cardiac hypertrophy, tumor growth, angiogenesis, mast-cell function, T-cell survival, sepsis, and synaptic plasticity and memory^14,24–28^. Constitutive germline genetic ablation of both *Rcan1* isoforms in the mouse confers resistance to abdominal AA (AAA), neointima formation, and atherosclerosis progression^14,26^. However, it has not been yet possible to ascribe specific roles to each Rcan1 isoform separately because previous studies have not selectively targeted *Rcan1-1* and *Rcan1-4*. Bone marrow transplantation experiments showed that while hematopoietic-cell expression of Rcan1 was not required for aneurysm^14^, it was critical for atherogenesis^26^. However, the individual contributions to these pathologies of smooth-muscle–expressed and endothelial-cell–expressed Rcan1 remain unexplored.

Also unknown are the molecular mechanisms through which Rcan1 contributes to these pathologies. Rcan1 was initially identified as an inhibitor of calcineurin activity^29^, but subsequent reports indicated that it can also activate calcineurin^30,31^. Moreover, our previous studies showed that Rcan1 does not regulate calcineurin activity in aortic tissues or in primary vascular smooth muscle cells (vSMCs) and macrophages^14,26^. It therefore seems that Rcan1 regulates vascular wall remodeling through interactions with proteins other than calcineurin.

Here, to gain insight into the role of Rcan1 in vascular disease, we use a proteomics approach to identify Rcan1-interacting proteins and engineer mice that enable tissue-specific inducible deletion of each isoform separately or both isoforms simultaneously. The resistance of constitutively *Rcan1*^*−/−*^ mice to vascular pathologies strongly suggested that strategies to inhibit RCAN1 expression or activity might be useful in the treatment of these diseases. However, we show here that the inducible deletion of *Rcan1* in SMCs or endothelial cells (ECs) disrupts aortic wall homeostasis, predisposing the aorta to hypertension-induced rupture, IMH, and aneurysm. Opposing effects are therefore observed in constitutive and inducible *Rcan1*-deficient mice.

## Results

### Induced *Rcan1* deletion predisposes to aortic rupture and IMH

**Fig. 1:**
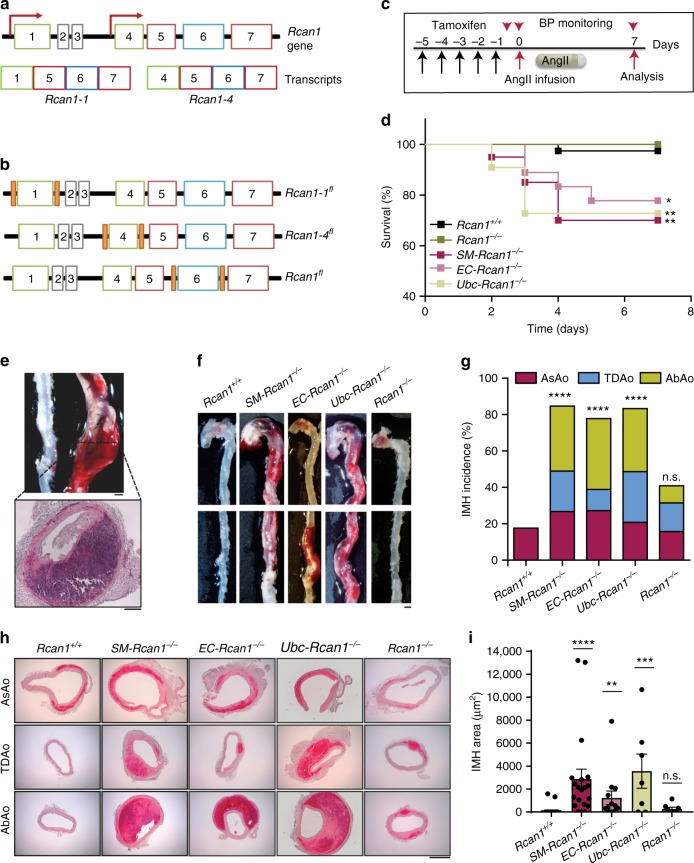
Inducible *Rcan1* deletion predisposes to aortic rupture and IMH. **a** Schematic representation of the *Rcan1* locus and *Rcan1* isoforms (Transcripts), indicating exons (boxes) and transcription initiation sites (arrows). **b** Relative position of LoxP sites (orange boxes) flanking exon 1, 4, or 6 in *Rcan1-1*^*fl/fl*^*, Rcan1-4*^*fl/fl*^, or *Rcan1*^*fl/fl*^, respectively. **c** Experimental design. Mice were treated with tamoxifen for 5 consecutive days (black arrows) before the implantation of osmotic minipumps for AngII infusion (1 μg kg^−1^ min^−1^). Mice were monitored for BP (red arrowheads) and euthanized after 7 days. **d** Survival curves of 6–8-week-old male *SM-Rcan1*^*−/−*^ (*n* = 20), *EC-Rcan1*^*−/−*^ (*n* = 18), *Ubc-Rcan1*^*−/−*^ (*n* = 11), *Rcan1*^*−/−*^ (*n* = 17), and *Rcan1*^*+/+*^ (*n* = 39) mice after AngII osmotic minipump implantation. Log-rank (Mantel-Cox) test, **p < 0.01, *p < 0.05 vs *Rcan1*^*+/+*^. All deaths were due to aortic rupture. **e** Representative macroscopic image (top) and hematoxylin-eosin stained section (bottom) of ruptured aortas from mice dead before the end of the experiment. Scale bars, 1 mm (top) and 200 µm (bottom). **f** Representative images of macroscopic hematomas in aortas from mice euthanized at the end of the experiment. Scale bar, 1 mm. **g** End-of-experiment IMH incidence in the AsAo, TDAo, and AbAo. Chi-square distribution, ****p < 0.0001 vs. *Rcan1*^*+/+*^; *n.s*., non-significant vs *Rcan1*^*+/+*^. **h** Representative images of aortic sections from *Rcan1*^*+/+*^, *SM-Rcan1*^*−/−*^, *EC-Rcan1*^*−/−*^, *Ubc-Rcan1*^*−/−*^, and *Rcan1*^*−/−*^ mice stained with hematoxylin-eosin. Scale bar, 500 µm. **i** Hemorrhage area in aortic sections. Each data point denotes an individual mouse, whereas histograms denote means ± s.e.m. Kruskal-Wallis with Dunn multiple comparison post-hoc test, ****p < 0.0001, ***p < 0.001, **p < 0.01 vs *Rcan1*^*+/+*^; *n.s*., non-significant vs. *Rcan1*^*+/+*^. **d**–**i** *Rcan1*^*+/+*^ littermates consisted of a pool of vehicle-treated Cre-positive and tamoxifen-treated Cre-negative *Rcan1*^*fl/fl*^ mice

To analyze the specific roles of Rcan1 isoforms in vascular wall remodeling, we generated inducible knockout mice specific for *Rcan1* isoforms. We used gene targeting to insert *LoxP* sites flanking *Rcan1* exon 1, exon 4, or exon 6 (Fig. 1a, b). Details of the targeting strategy are described in Supplementary Figure 1. Mice with LoxP-flanked *Rcan1* exon 1, exon 4, or exon 6 were crossed with mice expressing tamoxifen-inducible Cre recombinase (Cre^ERT2^) specifically in ECs (*Cdh5-Cre*^*ERT2*^)^32^ or SMCs (*Myh11-Cre*^*ERT2*^)^33^. Alternatively, mice with LoxP-flanked *Rcan1* exon 6 were crossed with mice expressing Cre^ERT2^ in a wide cell spectrum (*Ubc-Cre*^*ERT2*^)^34^. In this way, we were able to delete *Rcan1-1*, *Rcan1-4*, or both isoforms specifically in ECs (*EC*), SMCs (*SM*), or in most cells (*Ubc*) (Supplementary Table 1).

To confirm the specificity of the *Cdh5-Cre*^*ERT2*^ and *Myh11-Cre*^*ERT2*^ drivers, we generated *Myh11-Cre*^*ERT2*^;*Rosa26-LSL-YFP* mice and *Cdh5-Cre*^*ERT2*^;*Rosa26-LSL-Tomato* mice. Upon tamoxifen inoculation, *Myh11-Cre*^*ERT2*^;*Rosa26-LSL-YFP* mice expressed YFP only in the medial layer and *Cdh5-Cre*^*ERT2*^;*Rosa26-LSL-Tomato* mice expressed Tomato only in the intima (Supplementary Figure 2a, b). Transduction of vSMCs with GFP- or Cre-encoding lentivirus confirmed isoform-specific deletion within the *Rcan1* locus (Supplementary Figure 1e, f). To establish the isoform-specificity of the Cre-Lox system in the *Rcan1* locus in vivo, we treated *SM-Rcan1*^*fl/fl*^, *SM-Rcan1-1*^*fl/fl*^, and *SM-Rcan1-4*^*fl/fl*^ mice with tamoxifen and then induced Rcan1-4 expression by stimulation with AngII for 24 h (Supplementary Figure 2c). Immunoblot analysis of aortic protein extracts from these mice showed that deletion of exon 1, exon 4, or exon 6 specifically in SMCs markedly decreased aortic expression of Rcan1-1, Rcan1-4, or both isoforms (Supplementary Figure 2d). Protein expression was not completely lost, possibly because of the contribution of the intimal and adventitial layers. Efficient deletion of both *Rcan1* isoforms upon tamoxifen injection was confirmed in aortic tissue from *Ubc-Rcan1*^*−/−*^ mice (Supplementary Figure 2e).

While extracting the aortas of *SM-Rcan1*^*−/−*^ mice stimulated with AngII for 24 h, we occasionally observed pink shadows (Supplementary Figure 2f). Hematoxylin-eosin staining of aortic cross-sections showed the accumulation of blood in the medial layer without intimal tearing (Supplementary Figure 2g), indicating IMH. However, induced deletion of *Rcan1* isoforms caused no major symptoms in the absence of AngII: aortic tissue structure and values for body weight, heart rate, and blood pressure were not substantially different to those in *Rcan1*^*+/+*^ mice (Supplementary Figure 3), and no differences were observed in grooming, drinking, or eating habits.

To ascertain whether IMH was caused by the specific inactivation of *Rcan1* in SMCs, we deleted both *Rcan1* isoforms in ECs or SMCs and then treated the mice with AngII for 7 days (Fig. 1c). AngII significantly decreased survival relative to *Rcan1*^*+/+*^ littermates, which consisted of vehicle-treated Cre-positive or tamoxifen-treated Cre-negative *Rcan1*^fl/fl^ mice (Fig. 1d). Necropsy revealed the presence of aortic rupture and hemothorax or hemoabdomen (Fig. 1e). Nearly 90% of surviving AngII-treated conditional *Rcan1*^*−/−*^ mice had hematomas in the ascending aorta (AsAo), thoracic descending aorta (TDAo), or abdominal aorta (AbAo) (Fig. 1f, g). These results were unexpected because the constitutive deletion of both *Rcan1* isoforms protects against several aortic diseases^14,26^. In line with these earlier findings, constitutive *Rcan1*^*−/−*^ mice showed no predisposition to AngII-induced acute aortic rupture (Fig. 1d) and hematoma formation was not significantly more pronounced than in *Rcan1*^*+/+*^ C57BL/6 J mice (Fig. 1g). Histological analysis showed that all hematomas, including those found in the AbAo, consisted of bleeding within the medial layer in the absence of intimal tear or flap formation (Fig. 1h and Supplementary Figure 4). Quantification of the area covered by the hematoma in these sections showed that they were markedly larger in inducibly deficient mice than in constitutively deficient or *Rcan1*^*+/+*^ mice (Fig. 1i). A more detailed histological analysis of IMH in *SM-Rcan1*^*−/−*^ mice revealed an absence of collagen accumulation and the disorganization and rupture of the elastic lamellae (Supplementary Figure 4).

Tamoxifen priming of mice carrying the cardiomyocyte-specific *Cre*^*ERT2*^ cassette causes dose-dependent cardiac dysfunction within the first week following tamoxifen exposure and mitochondrial damage^35^. These results raised the possibility that Cre activation by tamoxifen, instead of *Rcan1* deletion, predisposed to IMH formation. However, none of the *Rcan1*^*+/+*^*;Myh11-Cre*^*ERT2*^, *Rcan1*^*+/+*^*;Cdh5-Cre*^*ERT2*^, and *Rcan1*^*+/+*^*;Ubc-Cre*^*ERT2*^ mice treated with tamoxifen and AngII died of aortic rupture or showed increased IMH incidence or extent relative to AngII-treated *Rcan1*^*+/+*^;Cre-negative mice (Supplementary Figure 5). Constitutive *Rcan1* depletion in cultured cardiomyocytes activates calcineurin and induces calcineurin-mediated mitochondrial fission^36^. In addition, *Rcan1-1* knockdown increases transcript levels for hexokinase 2 (*Hk2*)^36^. We therefore investigated whether constitutive or conditional *Rcan1* deletion induced mitochondrial aberrations in aortic smooth muscle cells. Flow cytometry analysis of cultured aortic smooth muscle cells from *SM-Rcan1*^*-/-*^, *Rcan1*^*-/-*^, or tamoxifen-treated *Rcan1*^*+/+*^*;Myh11-Cre*^*ERT2*^ mice stained with Mitotracker revealed no alteration of the mitochondrial content relative to Cre-negative *Rcan1*^*+/+*^ cells (Supplementary Figure 6a–c). Confocal microscope inspection of these cells showed that the number of mitochondria was also similar in all genotypes (Supplementary Figure 6d, e). Accordingly, the expression of *Hk2* and other mitochondrial genes remained unaltered (Supplementary Figure 6f). These data strongly suggest that no mitochondrial damage is caused by Cre activation or by constitutive or conditional *Rcan1* deletion in vSMCs.

Together, our results indicate that conditional and constitutive *Rcan1* inactivation cause opposite effects on aortic pathology. Since conditional *Rcan1* inactivation either in SMCs or in ECs predisposes to the same aortic lesion, our results also suggest that Rcan1 might be implicated in a cross-talk between both cell types necessary for aortic wall homeostasis maintenance.

**Fig. 2:**
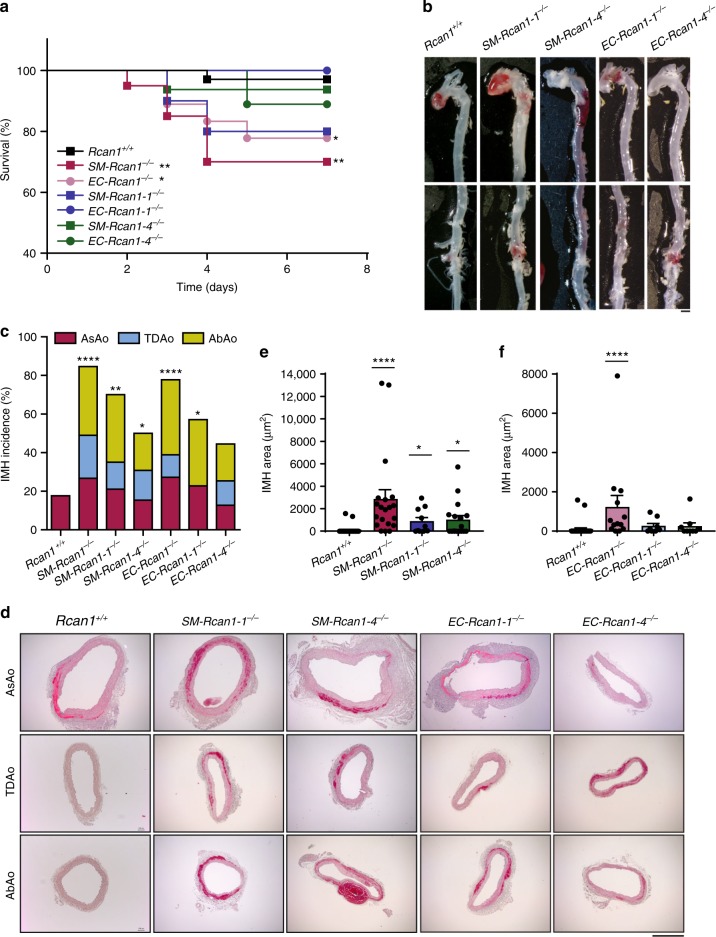
*Rcan1-1* and *Rcan1-*4 contribute to aortic homeostasis. Six-eight-week-old *SM-Rcan1-1*^*−/−*^ (*n* = 10), *SM-Rcan1-4*^*−/−*^ (*n* = 16), *EC-Rcan1-1*^*−/−*^ (*n* = 7), and *EC-Rcan1-4*^*−/−*^ (*n* = 9) male mice were treated with AngII at the same time as the *SM-Rcan1*^*−/−*^ (*n* = 20), *EC-Rcan1*^*−/−*^ (*n* = 18), and control *Rcan1*^*+/+*^ (*n* = 39) mice described in Fig. 1. Quantitative data for *SM-Rcan1*^*−/−*^, *EC-Rcan1*^*−/−*^, and control *Rcan1*^*+/+*^ are therefore the same in both figures. **a** Survival curves. Log-rank (Mantel-Cox) test, **p < 0.01, *p < 0.05 vs. *Rcan1*^*+/+*^. All deaths were due to aortic rupture. **b** Representative images of macroscopic hematomas in aortas from mice euthanized at the end of the experiment. Scale bar, 1 mm. **c** IMH incidence in the AsAo, TDAo, and AbAo of the same mice. Chi-square distribution, ****p < 0.0001, **p < 0.01, *p < 0.05 vs. *Rcan1*^*+/+*^. **d** Representative images of hematoxylin-eosin staining of aortic sections from the indicated genotypes and IMH area quantification in aortic sections from **e** *SM-Rcan1*^*−/−*^, *SM-Rcan1-1*^*−/−*^, and *SM-Rcan1-4*^*−/−*^ mice and **f** from *EC-Rcan1*^*−/−*^, *EC-Rcan1-1*^*−/−*^, *EC-Rcan1-4*^*−/−*^, and control *Rcan1*^*+/+*^ mice. Each data point denotes an individual mouse, whereas histograms denote means ± s.e.m. Kruskal-Wallis with Dunn multiple comparison post-hoc test, ****p < 0.0001, *p < 0.05 vs. *Rcan1*^*+/+*^. Scale bar, 500 µm. **a**–**d** *Rcan1*^*+/+*^ littermates consisted of a pool of vehicle-treated Cre-positive and tamoxifen-treated Cre-negative *Rcan1*^*fl/fl*^ mice

To determine the contribution of each Rcan1 isoform to aortic homeostasis, we treated *SM-Rcan1*^*−/−*^*, EC-Rcan1*^*−/−*^, *SM-Rcan1-1*^*−/−*^, *EC-Rcan1-1*^*−/−*^, *SM-Rcan1-4*^*−/−*^, EC*-Rcan1-4*^*−/−*^, and wt mice with AngII. Whereas simultaneous deletion of both *Rcan1* isoforms in either ECs or SMCs significantly increased the risk of aortic rupture upon exposure to AngII, this phenomenon was less frequent when only one isoform was deleted (Fig. 2a). Deletion of a single isoform promoted IMH in the presence of AngII (Fig. 2b), but the incidence was again lower than in mice with simultaneous deletion of both isoforms (Fig. 2c). Histological analysis also revealed smaller hematomas in mice lacking only one isoform than in mice lacking both (Fig. 2d–f). Together, these data suggest that Rcan1-1 and Rcan1-4 isoforms play similar and additive roles in the maintenance of aortic homeostasis.

### IMH evolves to AA or rupture in conditional *Rcan1*^*−/−*^ mice

**Fig. 3:**
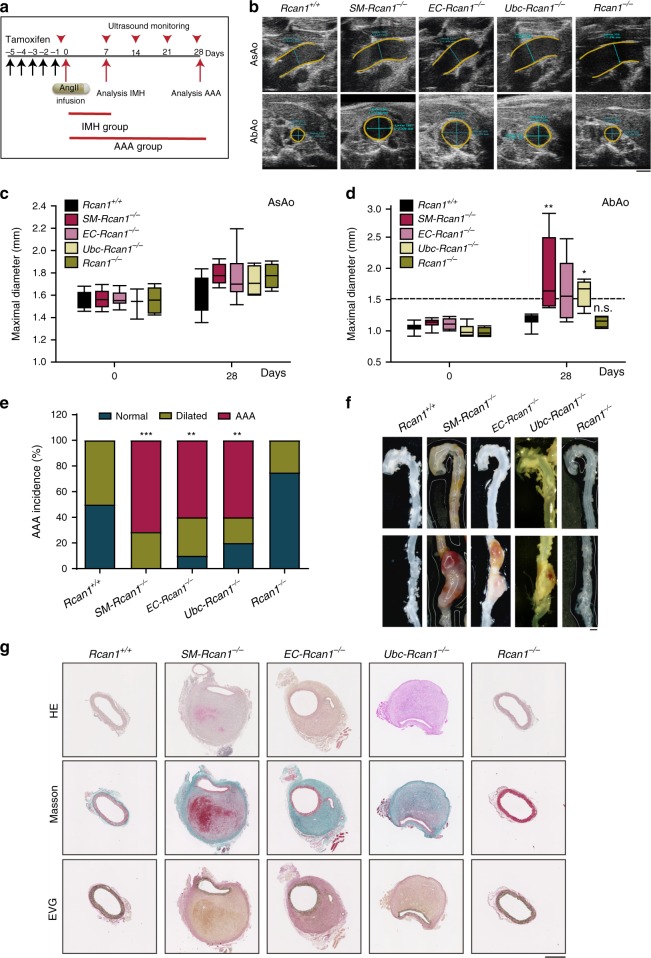
IMH progression into AAA in conditional *Rcan1*^*−/−*^ mice. **a** Experimental design: 6–8-week-old male mice were treated with tamoxifen for 5 consecutive days (black arrows) before the implantation of osmotic minipumps for AngII infusion (1 μg kg^−1^ min^−1^). Aortas were monitored by ultrasonography (red arrowheads), and mice were euthanized after 7 or 28 days of treatment with AngII. Data from the analysis performed after 7 days are shown in Supplementary Figure 7. **b** Representative ultrasound images of AsAo and AbAo from mice treated with AngII for 28 days. Yellow and blue lines mark the lumen boundary and the lumen diameter, respectively. Scale bar, 1 mm. Maximal (**c**) AsAo and (**d**) AbAo diameter at the indicated times of AngII treatment. The boxes represent the 25th and 75th percentile range of the mean values, the line in the box shows the median value, and the whiskers extend from the minimum to the maximum value. Two-way ANOVA with Tukey multiple comparison post-hoc test (**c**) and Kruskal-Wallis with Dunn multiple comparison post-hoc test (**d**), **p < 0.01, *p < 0.05 vs *Rcan1*^*+/+*^; n.s., non-significant vs. *Rcan1*^*+/+*^. **e** Incidence of AAA and aortic dilation. Normal, diameter < 1.2 mm; Dilated, 1.2 mm < diameter < 1.5 mm; AAA, diameter > 1.5 mm. Chi-square distribution, ***p < 0.001, **p < 0.01, vs. *Rcan1*^*+/+*^. **f** Representative images of AAA. Scale bar, 1 mm. **g** Representative images of aortic sections stained with hematoxylin-eosin (HE), Masson’s trichrome (Masson) and Elastic Van Gieson stain (EVG). Scale bar, 500μm. *Rcan1*^*+/+*^ (*n* = 12), *SM-Rcan1*^*−/−*^ (*n* = 7), *EC-Rcan1*^*−/−*^ (*n* = 11), *Ubc-Rcan1*^*−/−*^ (*n* = 6), and *Rcan1*^*−/−*^ (*n* = 6). **b**–**g** *Rcan1*^*+/+*^ littermates consisted of a pool of vehicle-treated Cre-positive and tamoxifen-treated Cre-negative *Rcan1*^*fl/fl*^ mice

The most frequent outcome of the long-term clinical progression of IMH is aortic aneurysm or pseudoaneurysm^1^. To determine the development of IMH in *Rcan1*-deficient mice, we used ultrasound imaging to monitor for the presence of aneurysms in the aortas of mice treated with AngII for 28 days. We also conducted postmortem examinations in a parallel group of mice treated with AngII for 7 days (Fig. 3a). In line with the results shown in Fig. 1, nearly 40% of AngII-infused *SM-Rcan1*^*−/−*^, *EC-Rcan1*^*−/−*^, and *Ubc-Rcan1*^*−/−*^ mice died within the first week of treatment, whereas 100% of constitutive *Rcan1*^*−/−*^ mice survived (Supplementary Figure 7a). In addition, >80% of *SM-Rcan1*^*−/−*^, *EC-Rcan1*^*−/−*^, and *Ubc-Rcan1*^*-/-*^ mice sacrificed after 7 days’ treatment with AngII showed IMH in the AsAo and/or the TDAo or the AbAo (Supplementary Figure 7b–e). Treatment of conditional or constitutive *Rcan1*^*-/-*^ mice with AngII for 28 days did not substantially enlarge AsAo diameter relative to *Rcan1*^*+/+*^ mice (Fig. 3b, c). However, this treatment sharply increased AbAo diameter in *SM-Rcan1*^*−/−*^, *EC-Rcan1*^*−/−*^, and *Ubc-Rcan1*^*−/−*^ mice relative to *Rcan1*^*+/+*^ mice, but not in constitutive *Rcan1*^*−/−*^ mice (Fig. 3b, d). In fact, 100% of conditional *Rcan1*^*−/−*^ mice showed either abdominal aortic aneurysm (AAA) or dilated AbAo (diameter = 1.2–1.5 mm) upon AngII infusion (Fig. 3e). AAA in these mice was suprarenal (Fig. 3f) and featured marked collagen deposition and destabilization of the ordered multilayered vessel wall structure (Fig. 3g). In contrast, constitutive *Rcan1*^*−/−*^ mice showed even less dilatation than *Rcan1*^*+/+*^ mice and the aortic wall structure in these genotypes was indistinguishable (Fig. 3e–g). Together, these data suggest that while AngII-induced hemorrhages in the AsAo either progress to aortic rupture or regress, IMH in the AbAo progresses to aortic rupture or to AA.

**Fig. 4:**
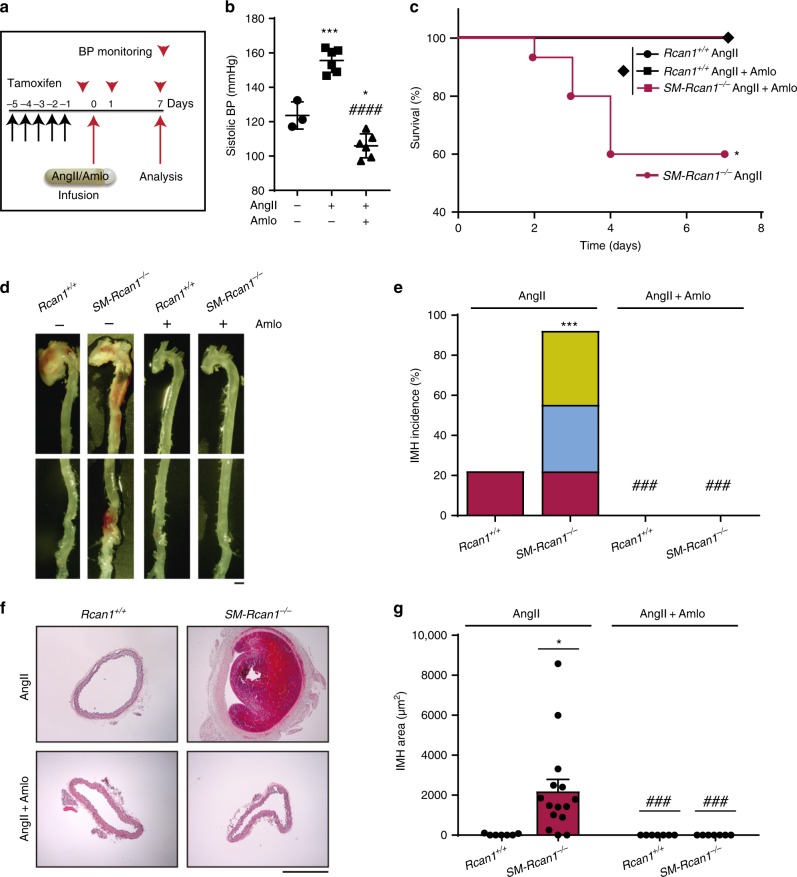
The hypertensive effect of AngII is required for IMH formation. **a** Experimental design: 6–8-week-old male mice were treated with tamoxifen for 5 consecutive days (black arrows) before the implantation of osmotic minipumps for infusion of AngII (1 μg kg^−1^ min^−1^) and amlodipine (Amlo, 6 mg kg^−1^ day^−1^). Mice were monitored for blood pressure (BP; red arrowheads) and euthanized at the end of the experiment. **b** Systolic BP measurements in *Rcan1*^*+/+*^ mice treated with saline (*n* = 3), AngII (*n* = 6), or Amlo plus AngII (*n* = 6). One-way ANOVA with Tukey multiple comparison post-hoc test, ***p < 0.001, *p < 0.05 vs. untreated, ^###^*p* < 0.001 vs. AngII. **c** Survival curve of *Rcan1*^*+/+*^ and *SM-Rcan1*^*−/−*^ mice treated with AngII and Amlo as indicated. Log-rank (Mantel-Cox) test, *p < 0.05 vs. *Rcan1*^*+/+*^ AngII. All deaths were due to aortic rupture. **d** Representative images of macroscopic hematomas in aortas from mice treated with AngII or AngII + Amlo (scale bar, 1 mm) and **e** hematoma incidence in these mice. Chi-square distribution, ***p < 0.001 vs. *Rcan1*^*+/+*^ AngII, ^###^*p* < 0.001 vs. *SM-Rcan1*^*−/−*^ AngII. **f** Hematoxylin-eosin staining of AbAo cross-sections from the same mice (scale bar, 500 µm) and **g** IMH area quantification in these sections shown as mean + s.e.m; each data point denotes an individual mouse. *Rcan1*^*+/+*^ AngII (*n* = 8), *Rcan1*^*+/+*^ AngII + Amlo (*n* = 6), *SM-Rcan1*^*−/−*^ AngII (*n* = 15), and *SM-Rcan1*^*−/−*^ AngII + Amlo (*n* = 6). Kruskal-Wallis with Dunn multiple comparison post-hoc test, *p < 0.05 vs. *Rcan1*^*+/+*^ AngII, ###*p* < 0.001 vs *SM-Rcan1*^*−/−*^ AngII. **c**–**g** *Rcan1*^*+/+*^ littermates consisted of a pool of vehicle-treated Cre-positive and tamoxifen-treated Cre-negative *Rcan1*^*fl/fl*^ mice

AngII is best known for its hypertensive action, but AngII is also implicated in processes related to pathological vessel remodeling, including fibrosis, inflammation, and vascular aging^37–39^. To test whether AngII induced IMH through its hypertensive effect, we treated *SM-Rcan1*^*−/−*^ mice simultaneously with AngII and amlodipine (Fig. 4a), an established antihypertensive agent^40^. Amlodipine blocked not only AngII-induced hypertension (Fig. 4b), but also the occurrence of lethal aortic ruptures (Fig. 4c) and IMH along the aorta (Fig. 4d–g). We also treated *SM-Rcan1*^*−/−*^ mice simultaneously with AngII and with the combination of the vasorelaxant hydralazine plus hydrochlorothiazide. Similar to amlodipine, this vasodilator treatment prevented aortic rupture and blocked AngII-induced hypertension and IMH formation (Supplementary Figure 8). To further confirm the role of hypertension in IMH formation, we treated *SM-Rcan1*^*−/−*^ mice with the vasopressor norepinephrine. A norepinephrine dose equi-prohypertensive to AngII induced IMH formation in SM-*Rcan1*^*−/−*^ mice as efficiently as AngII (Supplementary Figure 9). Norepinephrine, however, did not induce aortic rupture in conditional *Rcan1*^*−/−*^ mice (Supplementary Figure 9b), suggesting that AngII-induced hypertension is necessary but not sufficient to elicit aortic rupture. Together, these data strongly suggest that conditional Rcan1 deficiency alters vascular wall homeostasis to predispose the aorta to AngII-induced pathological vessel remodeling.

### *SM-* and *EC-Rcan1* deficiency increases vascular permeability

**Fig. 5:**
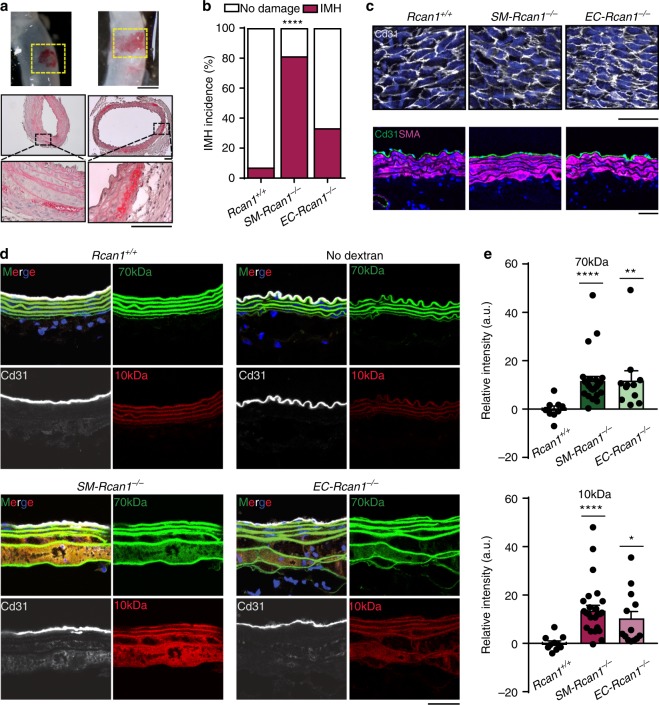
Conditional *Rcan1*^*−/−*^ mice show increased aortic permeability. **a** (Top panels) Representative images of macroscopic incipient hematomas found after 6 h of AngII infusion (1 μg kg^−1^ min^−1^). Scale bar, 1 mm. (Middle and bottom panels) Hematoxylin-eosin staining of these hematomas. Scale bars, 100 µm. **b** Incidence of aortic IMH after 6 h of AngII infusion in *Rcan1*^*+/+*^ (*n* = 14), *SM-Rcan1*^*−/−*^ (*n* = 16), and *EC-Rcan1*^*−/−*^ mice (*n* = 12). Chi-square distribution, ****p < 0.0001 vs. *Rcan1*^*+/+*^. **c** Representative images of Cd31 immunofluorescence in en face (top panels) and transversal section (bottom panels) of aortas from mice infused for 6 h with AngII. Cd31 (gray, top panels; green, bottom panels), DAPI-stained nuclei (blue). Scale bar, 20 μm. **d** Representative images showing the accumulation of FITC-labeled 70-kDa dextran (green) and RhodamineB-labeled 10-kDa dextran (red) in aortas of *Rcan1*^*+/+*^ (*n* = 10), *SM-Rcan1*^*−/−*^ (*n* = 16), and *EC-Rcan1*^*−/−*^ (*n* = 12) mice infused for 6 h with AngII. Elastin autofluorescence is shown also in green. Aortas were also stained with anti-Cd31 (gray) and DAPI (blue). Scale bar, 50 µm. **e** Fluorescence intensity quantification of the accumulation of (top) FITC-labeled 70-kDa dextran and (bottom) RhodamineB-labeled 10-kDa dextran in aortic sections from the same mice; a.u., arbitrary units. Each data point denotes an individual mouse, whereas histograms denote means + s.e.m. Kruskal-Wallis with Tukey Dunn multiple comparison post-hoc test, ****p < 0.0001, **p < 0.01, *p < 0.05 vs *Rcan1*^*+/+*^. **b**–**e** *Rcan1*^*+/+*^ littermates consisted of a pool of vehicle-treated Cre-positive and tamoxifen-treated Cre-negative *Rcan1*^*fl/fl*^ mice

We investigated whether AngII induced IMH formation early after its infusion. A 6 h AngII treatment induced incipient IMH in 80% of *SM-Rcan1*^*−/−*^, 33% of *EC-Rcan1*^*−/−*^ mice, and 7% of *Rcan1*^*+/+*^ mice (Fig. 5a, b). Hematoxilin-eosin staining of these inceptive lesions showed that hemorrhages were contained in the outer lamellar units of the medial layer, but never close to the intimal layer (Fig. 5a). Immunostaining of aortic ECs in whole-mount or transverse sections from *SM-Rcan1*^*−/−*^ and *EC-Rcan1*^*−/−*^ mice showed intimal layer integrity both in saline-treated mice (Supplementary Figure 10a, b) and in AngII-treated mice (Fig. 5c), suggesting that the bleeding came from adventitial or from peri-aortic vessels.

To identify the point of blood entry into the tunica media, we injected 10-kDa and 70-kDa fluorescently labeled dextrans into the tail veins of *Rcan1*^*+/+*^, *SM-Rcan1*^*−/−*^, and *EC-Rcan1*^*−/−*^ mice treated for 6 h with AngII. In the aorta, labeled dextrans were detected only in the outer lamellar units of the media (Fig. 5d, e). Immunostaining of ECs showed no intimal layer disruption in the region where dextrans were found (Fig. 5d). A search for labeled dextrans in highly vascularized tissues revealed a modest increase of 10-kDa dextrans in the kidneys of *SM-Rcan1*^*−/−*^ and *EC-Rcan1*^*−/−*^ mice, but not in the lungs (Supplementary Figure 11a–c). No hemorrhages were found in these tissues (Supplementary Figure 11d).

### ROCK-mediated MLC activation predisposes to aortic disease

**Fig. 6:**
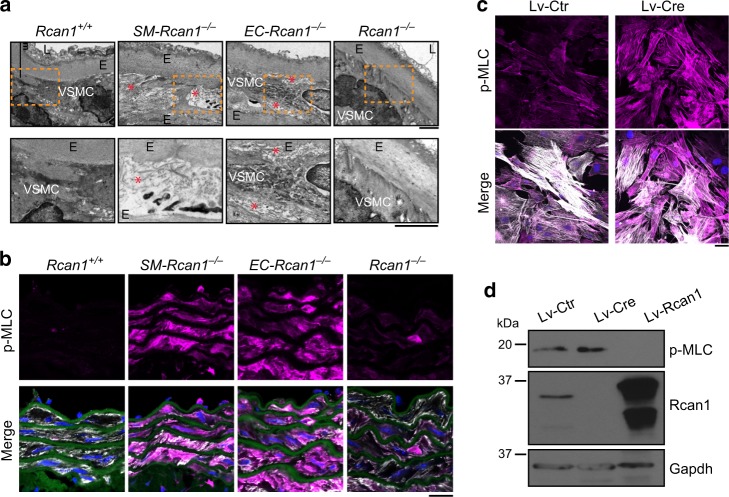
Vascular ultrastructure alteration and MLC activation in inducible *Rcan1*^*−/−*^ mice. **a** Representative transmission electron microscopy (TEM) images of aortic sections from *Rcan1*^*+/+*^ (*n* = 6), *SM-Rcan1*^*−/−*^ (*n* = 6), *EC-Rcan1*^*−/−*^ (*n* = 6), and *Rcan1*^*−/−*^ mice (*n* = 4). L, lumen; E, elastic lamella; vSMC, vascular smooth muscle cells. Red asterisks mark regions with spaces between elastic layers and vSMCs. Bar, 2 µm. **b** Representative images of elastin autofluorescence (green), DAPI-stained nuclei (blue), and p-MLC (pink) and SMA (gray) immunofluorescence on aortic sections from *Rcan1*^*+/+*^ (*n* = 6), *SM-Rcan1*^*−/−*^ (*n* = 6), *EC-Rcan1*^*−/−*^ (*n* = 6), and *Rcan1*^*−/−*^ mice (*n* = 11). Scale bar, 20 µm. **c** Representative images of DAPI staining (blue), and p-MLC (pink) and SMA (gray) immunofluorescence in *Rcan1*^*fl/fl*^ vSMCs transduced with Cre-encoding or control lentiviral vectors. Scale bar, 20 µm. **d** Representative p-MLC, Rcan1, and tubulin immunoblots of *Rcan1*^*fl/fl*^ vSMCs transduced with Cre-encoding or control lentivirus and treated with the ROCK inhibitor Fasudil (30 µM) for 1 h (*n* = 3 independent experiments). **a**, **b** *Rcan1*^*+/+*^ littermates consisted of a pool of vehicle-treated Cre-positive and tamoxifen-treated Cre-negative *Rcan1*^*fl/fl*^ mice

The rapid onset of IMH formation upon AngII-induced acute hypertension suggests that aortic tissue in conditionally *Rcan1*-deficient mice may have underlying structural defects that are exacerbated by AngII. Transmission electron microscopy of *SM-Rcan1*^*−/−*^ and *EC-Rcan1*^*−/−*^ aortas revealed loss of vSMC contact with the elastic lamina, a markedly increased intercellular space, and extracellular accumulation of microfibers (Fig. 6a). The decreased area occupied by vSMCs suggested that they might be undergoing apoptosis or be in a more contractile state than in *Rcan1*^*+/+*^ or constitutive *Rcan1*^*−/−*^ mice. Staining of aortic sections with ApopTag confirmed the absence of apoptotic cells in *SM-Rcan1*^*−/−*^ and *Rcan1*^*+/+*^ mice (Supplementary Figure 12a). Examination of markers of vSMC contractile phenotype also revealed no differences in *Acta2*, *Myh11*, and *Cnn1* mRNA content (Supplementary Figure 12b). However, aortas from *SM-Rcan1*^*−/−*^ and *EC-Rcan1*^*−/−*^ mice showed substantially higher phosphorylation of myosin light chain (MLC) (Fig. 6b), a regulatory subunit whose phosphorylation promotes myosin-mediated contractility^41^. We confirmed these data in aortic smooth muscle cells isolated from *Rcan1*^*fl/fl*^ mice; transduction of these cells with Cre-encoding lentiviral vectors markedly increased p-MLC staining relative to cells transduced with a control lentivirus (Fig. 6c). Accordingly, forced Rcan1 expression had the opposite effect, decreasing p-MLC levels (Fig. 6d). Of note, aortas from *Rcan1*^*−/−*^ mice showed no substantial increase of MLC phosphorylation (Fig. 6b), suggesting that compensatory mechanisms might act during embryonic development or early post-natal stages to prevent MLC hyperphosphorylation.

**Fig. 7:**
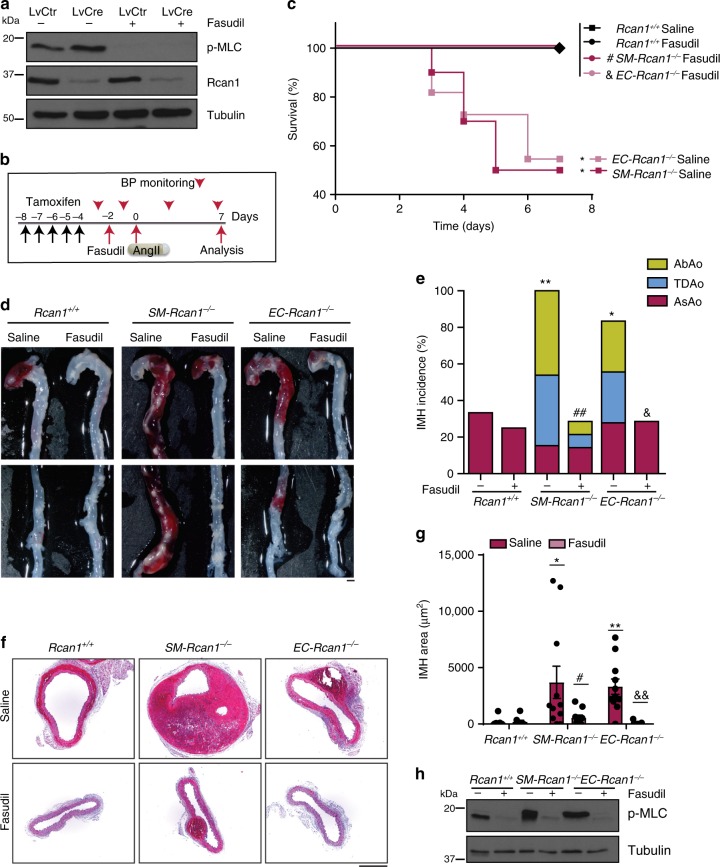
ROCK mediates MLC activation and aortic disease. **a** Representative p-MLC, Rcan1, and tubulin immunoblots of *Rcan1*^*fl/fl*^ vSMCs transduced with Cre-encoding or control lentivirus and treated with the ROCK inhibitor Fasudil (30 µM) for 1 h (*n* = 3 independent experiments). **b** Experimental timeline. Black arrows, tamoxifen injection. A group of mice was treated with Fasudil (1 mg per ml in drinking water) starting 2 days before 7-day treatment with AngII. Red arrowheads, BP measurements. Red arrows, Fasudil start of treatment (−2), AngII osmotic minipump implantation (0), and end-of-experiment (7). **c** Survival curves of 6–8-week-old male mice treated according to the scheme in **b** as indicated. Saline = AngII-treated: *Rcan1*^*+/+*^ (*n* = 7), *SM-Rcan1*^*−/−*^ (*n* = 10), and *EC-Rcan1*^*−/−*^ (*n* = 11); Fasudil = Fasudil + AngII treated: *Rcan1*^*+/+*^ (*n* = 8), *SM-Rcan1*^*−/−*^ (*n* = 7), and *EC-Rcan1*^*−/−*^ (*n* = 11). Log-rank (Mantel-Cox) test, *p < 0.05 vs *Rcan1*^*+/+*^ AngII (Saline), #*p* < 0.05 vs. *SM-Rcan1*^*−/−*^ AngII (Saline), &*p* < 0.05 vs. *EC-Rcan1*^*−/−*^ AngII (Saline). All deaths were due to aortic rupture. **d** Representative images of aortas with macroscopic hematomas (scale bar, 1 mm) and **e** hematoma incidence in these mice. Chi-square distribution, *p < 0.05, **p < 0.01, vs. *Rcan1*^*+/+*^ Saline, ##*p* < 0.01 vs. *SM-Rcan1*^*−/−*^ Saline, *p* < 0.05 vs. *EC-Rcan1*^*−/−*^ Saline. **f** Representative images of HE staining in AbAo sections from these mice (scale bar, 500 µm) and **g** IMH area quantification in these sections shown as mean ± s.e.m.; each data point denotes an individual mouse. Kruskal-Wallis with Tukey Dunn multiple comparison post-hoc test, **p < 0.01, *p < 0.05 vs. *Rcan1*^*+/+*^ Saline, #*p* < 0.05 vs *SM-Rcan1*^*−/−*^ Saline, &&*p* < 0.01 vs *EC-Rcan1*^*−/−*^ Saline. **h** Representative p-MLC immunoblot of aortic extracts from the same mice. Tubulin was used as loading control. **c**–**h** *Rcan1*^*+/+*^ littermates consisted of a pool of vehicle-treated Cre-positive and tamoxifen-treated Cre-negative *Rcan1*^*fl/fl*^ mice

MLC phosphorylation can be induced upon inactivation of MLC phosphatase mediated by RhoA kinase (ROCK)^42^. We found that the ROCK inhibitor Fasudil^43^ blocked MLC phosphorylation in *Rcan1*^*fl/fl*^ aortic SMCs transduced with a control lentivirus or Cre-encoding lentivirus (Fig. 7a). Furthermore, Fasudil blocked AngII-induced mortality in *SM-Rcan1*^*−/−*^, *EC-Rcan1*^*−/−*^, and control mice (Fig. 7b, c) and sharply decreased IMH incidence along the aorta and IMH size in conditional *Rcan1*^*−/−*^ mice (Fig. 7d–g). Immunoblot and immunofluorescence analysis revealed that Fasudil substantially decreased MLC phosphorylation in the aortas of these mice (Fig. 7h and Supplementary Figure 13a). Notably, the protective effect of Fasudil was independent of blood pressure regulation (Supplementary Figure 13b). Together, these data indicate that, contrary to constitutive *Rcan1* genetic inactivation, its conditional deletion promotes a hypercontractile phenotype in aortic SMCs that predisposes to aortic rupture and IMH.

### Gsk-3 β mediates MLC activation in conditional *Rcan1*^*−/−*^ mice

**Fig. 8:**
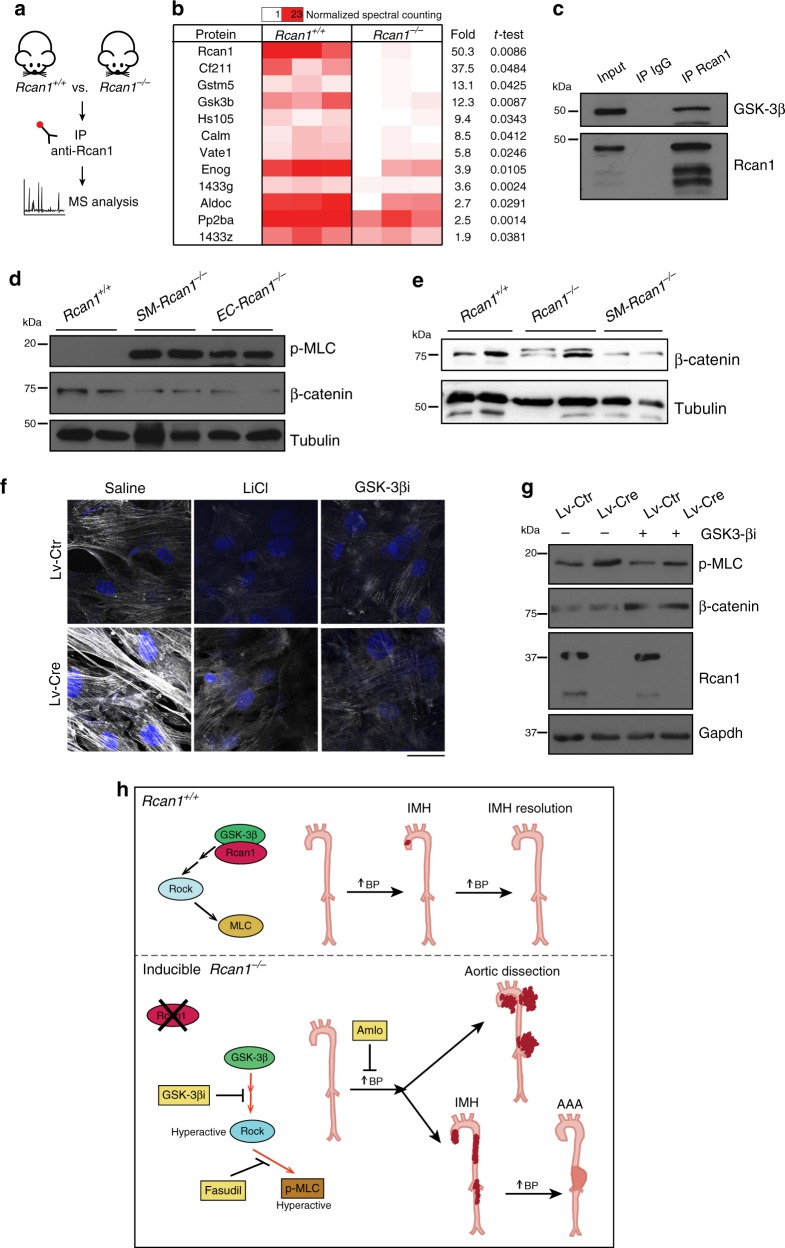
GSK-3 β mediates MLC activation in inducible *Rcan1*^*−/−*^ mice. **a** Experimental design. Protein extracts from brains of *Rcan1*^*+/+*^ and *Rcan1*^*−/−*^ mice were immunoprecipitated with anti-Rcan1 antibodies, and interacting proteins were identified by mass spectrometry and quantified by spectral counting. **b** Significantly enriched proteins (Student *t*-test, *p* < 0.05) identified in Rcan1 immunoprecipitates from *Rcan1*^*+/+*^ and *Rcan1*^*−/−*^ extracts are listed according to their enrichment in 3 independent experiments. **c** Gsk-3 β and Rcan1 immunoblot analysis of vSMC protein extracts immunoprecipitated with anti-Rcan1 antibody (IP Rcan1) or control antibody (IP IgG), compared with crude extract (Input). **d** Representative p-MLC, β-catenin, and tubulin (loading control) immunoblot analysis of aortic extracts from *Rcan1*^*+/+*^, *SM-Rcan1*^*−/−*^, and *EC-Rcan1*^*−/−*^ mice (*n* = 3 independent experiments). **e** Representative β-catenin, and tubulin (loading control) immunoblot analysis of aortic extracts from *Rcan1*^*+/+*^ (*n* = 7), *Rcan1*^*−/−*^ (*n* = 6) and *SM-Rcan1*^*−/−*^ (*n* = 8) mice. **d**, **e** *Rcan1*^*+/+*^ littermates consisted of a pool of vehicle-treated Cre-positive and tamoxifen-treated Cre-negative *Rcan1*^*fl/fl*^ mice. **f** Representative images (*n* = 3) of p-MLC immunofluorescence (gray) and DAPI staining (blue) in *Rcan1*^*fl/fl*^ vSMCs transduced with Cre-encoding or control lentivirus and treated for 2 h with saline or the GSK-3 β inhibitors LiCl (50 µM) or inhibitor VIII (GSK-3 βi) (10 µM). Scale bar, 20 µm. **g** Representative Rcan1, p-MLC, and β-catenin immunoblot analysis (*n* = 3) in vSMCs from **f**. Gapdh was used as loading control. **h** Model depicting the GSK-3 β- and ROCK-mediated induction of aortic IMH, aortic dissection, and AAA in conditional *Rcan1*^*−/−*^ mice subjected to hypertensive stress. While hypertension induces spontaneously-regressing small IMH in *Rcan1*^*+/+*^ mice at low frequency, in most conditional *Rcan1*^*−/−*^ mice it induces large IMH that dissect or progress to aneurysm

To investigate the molecular mechanisms involved in MLC activation upon inducible *Rcan1* deletion, we conducted a proteomics analysis of Rcan1-interacting proteins. The low amount of Rcan1 in the aorta precluded use of this organ, and we therefore used brain extracts, in which Rcan1 is abundant. Rcan1-interacting proteins were identified by mass spectrometry of Rcan1 immunoprecipitates from *Rcan1*^*+/+*^ and *Rcan1*^*−/−*^ mouse brain extracts (Fig. 8a). Rcan1 was specifically immunoprecipitated from *Rcan1*^*+/+*^ mice extracts together with several potential Rcan1-interacting candidates, one of the most significantly enriched being Gsk-3 β (Fig. 8b). GSK-3 β is a candidate activator of ROCK^44,45^, suggesting that Rcan1 might regulate Gsk-3 β-mediated activation of ROCK and MLC. Immunoblot analysis confirmed the Rcan1–Gsk-3 β association in vSMCs (Fig. 8c). As a readout of Gsk-3 β activity in aorta, we measured the protein content of β-catenin, which is degraded upon Gsk-3 β-mediated phosphorylation^46,47^. β-catenin levels were lower in *SM-Rcan1*^*−/−*^ and *EC-Rcan1*^*−/−*^ aorta than in *Rcan1*^*+/+*^ aorta (Fig. 8d). In contrast, p-MLC levels were markedly elevated in both inducibly Rcan1-deficient genotypes (Fig. 8d). Contrary to induced *Rcan1* deletion, its constitutive inactivation did not substantially affect β-catenin levels (Fig. 8e), suggesting that compensatory mechanisms prevented Gsk-3 β activation in the aorta of constitutive *Rcan1*^*−/−*^ mice. Gsk-3 β inhibition with lithium chloride^48^ or the highly specific Gsk-3 β inhibitor VIII^49^ blocked the MLC phosphorylation induced upon *Rcan1* deletion in vSMCs from *Rcan1*^*fl/fl*^ mice (Fig. 8f, g). Consistent with the role of Gsk-3 β in β-catenin proteolysis, Gsk-3 β inhibitor VIII prevented β-catenin degradation in parallel control experiments (Fig. 8g).

## Discussion

Our previous studies in constitutive *Rcan1*^*−/−*^ mice showed that Rcan1 is a critical mediator of neointima formation, aneurysm, and atherosclerosis^14,26^. Here, we used mice with tissue-specific inducible deletion of *Rcan1* isoforms to gain insight into the role of *Rcan1* isoforms in vascular pathologies. Unexpectedly, we found that inducible and constitutive deletion of *Rcan1* produce opposing effects in the aorta: inducible *Rcan1* deletion in *Apoe*^*+/+*^ mice but not its constitutive deletion predisposes to AngII-induced lethal aortic rupture and IMH. It is important to note that we analyzed constitutive and inducible *Rcan1*^*−/−*^ mice in parallel. Our results strongly support the notion that Rcan1 plays a critical homeostatic role in the aorta and that compensatory mechanisms are induced during embryonic development or early post-natal stages of constitutive *Rcan1*^*−/−*^ mice. Furthermore, our data strongly suggest that interaction with Rcan1 limits Gsk-3 β activity and therefore the activation of the ROCK/MLC axis under homeostatic conditions. Disruption of this pathway in inducible *Rcan1*^*−/−*^ mice severely compromises arterial wall homeostasis through the induction of a contractile vSMC phenotype. However, this pathway was preserved in constitutive *Rcan1*^*−/−*^ mice, suggesting that compensatory mechanisms might prevent Gsk-3 β activation during development in these mice. Inducing *Rcan1* deletion during different developmental stages might help to elucidate this issue and uncover the mechanisms of compensation.

Although IMH is a severe condition that can progress to aortic dissection or aortic aneurysm, very little is known about its etiology and the molecular mechanisms underlying its formation. We show that AngII elicited IMH in the AsAo of <20% wt mice, and these hematomas were small. These results are in line with those reported by Rateri et al.^10^. It is important to note that these hematomas very rarely caused aortic rupture and we never detected their progression to aneurysm. Similarly, AngII also induced small IMH in constitutive *Rcan1*^*−/−*^ mice, and these hematomas also did not progress either to aneurysm or to aortic rupture. In contrast, AngII infusion into conditional *Rcan1*^*−/−*^ mice readily induced lethal aortic rupture and IMH that progressed to aneurysm. Since the phenotype of AngII-treated conditional *Rcan1*^*−/−*^ mice resembles human IMH disease, these mice appear to provide a preclinical model for investigating the mechanisms underlying IMH and testing candidate therapies.

In human IMH, it is unclear if the blood comes from the aortic lumen or a leaky vasa vasorum. We were unable to identify the blood entry point in our mouse models. However, as in the human disease, the aortic intimal layer was always intact in the hematoma region. In addition, incipient hematomas detected early after AngII administration were always located in the outer elastic lamellar units. Since there is no vasa vasorum in the nonatherosclerotic mouse aorta^50^, our results suggest that the blood might leak from adventitial small vessels or periaortic vessels. We might therefore expect blood to leak from other small vessels in these mice, but did not detect hemorrhages in any other organ. However, fluorescently labeled 10-kDa dextrans were extravasated to the kidney, suggesting that AngII increased the permeability of small vessels in *Rcan1*-deficient mice. These results are in line with a recent report showing increased vessel permeability in *Rcan1*^*−/−*^ mice during the anaphylactic response^51^. We hypothesize that AngII might increase vascular permeability of various small vessels in conditional *Rcan1*^*−/−*^ mice and that while the body’s repair mechanisms may have no access to the blood extravasated into the outer laminae of the aorta, these mechanisms might readily remove blood leaked into other organs.

Clinical IMH is associated with hypertension^2–4^, and the vasopressor AngII induces IMH in the mouse, suggesting that increased blood pressure might be a cause of IMH. Hypertension is also a major risk factor for aortic aneurysm and dissection in humans^5–7^. Although AngII also induces aneurysm and aortic rupture in the mouse, the contribution of AngII-induced hypertension to these conditions is controversial, as opposing results were reported in normocholesterolemic and hypercholesterolemic mice^12,52^. AngII also promotes inflammation and induces fibrosis^37–39^, two events involved in pathological vessel remodeling. H&E staining of incipient IMH in the mouse did not reveal an inflammatory response in the aorta, and we did not detect substantial collagen accumulation in this organ, suggesting that the AngII-dependent IMH induction was caused by the acute increase in blood pressure. Supporting this hypothesis, amlodipine, a well-known hypotensive agent, blocked IMH formation in our mouse models. Similarly, the hypotensive combination of hydralazine plus hydrochlorothiazide also blocked IMH formation. Further supporting our hypothesis, the vasopressor norepinephrine increased IMH formation. It seems therefore likely that AngII induces IMH and aneurysm through its hypertensive capacity in normocholesterolemic mice and through hypertension-independent mechanisms in hypercholesterolemic mice. While amlodipine or the combination of hydralazine plus hydrochlorotiazide also prevented AngII-induced aortic rupture in conditional *Rcan1*^*−/−*^ mice, norepinephrine infusion of these mice did not induce aortic rupture, suggesting that AngII-induced hypertension is necessary but not sufficient to elicit aortic rupture.

Conditional *Rcan1-1*^*−/−*^ and *Rcan1-4*^*−/−*^ mice showed a predisposition to form IMH following AngII administration, but the effect was less marked upon deletion of a single isoform than in mice simultaneously lacking both isoforms. This suggests that the two isoforms play a similar and additive role in the maintenance of aortic homeostasis. Our data also show that aortic homeostasis depends on Rcan1 expression in both SMCs and ECs, because its specific deletion in either cell type predisposed to lethal aortic dissection and IMH formation. This result suggests that aortic wall homeostasis involves crosstalk between vSMCs and ECs. In this regard, our ultrastructural analysis of the aortic wall indicated that *SM-Rcan1*^*−/−*^ and *EC-Rcan1*^*−/−*^ aortas have similar medial-layer defects before AngII administration: substantial enlargement of the intercellular space, accumulation of extracellular matrix, and partial loss of vSMC contact with elastic fibers. The increased intercellular space was consistent with a hypercontractile vSMC state or the induction of apoptosis. While no apoptotic cells were found in the aorta of these mice, there was a marked increase in p-MLC, which activates myosin-dependent contractile force. Our results thus suggest that conditional Rcan1 loss in ECs or in vSMCs induces a hypercontractile phenotype in vSMCs.

MLC phosphorylation is dynamically regulated by MLC kinase (MYLK) and MLC phosphatase (MLCP), whose activities are controlled through several pathways, including the inactivating phosphorylation of MLCP by ROCK (reviewed in^53^). Our finding that the ROCK inhibitor Fasudil inhibits MLC phosphorylation and sharply inhibits the occurrence of lethal aortic rupture and IMH strongly suggests that conditional *Rcan1* inactivation leads to ROCK-mediated MLC activation. Although we cannot exclude the possibility that MYLK becomes activated in the aortas of conditional *Rcan1*^*−/−*^ mice, our results are in line with a recent report showing the involvement of ROCK in the regulation of vascular permeability^54^.

Rcan1 can activate or repress CN activity^29–31^. Rcan1 depletion in cultured cardiomyocytes, in particular, activates CN and induces CN-mediated mitochondrial fission^36^. However, we found no mitochondrial aberrations in constitutive or in conditional *Rcan1*^*−/−*^ vSMCs. These results suggest that *Rcan1* depletion in vSMCs does not affect CN activity and are therefore in line with our previous observations showing that CN activity was unaffected by Rcan1 expression in aortic tissue, vSMCs, or macrophages^14,26^. It therefore seems likely that Rcan1 regulates distinct signaling pathways in cardiomyocytes and vascular cells. Our proteomics analysis identified Gsk-3 β as a potential Rcan1-interacting protein. Immunoprecipitation/immunoblotting confirmed interaction between these proteins in aortic SMCs, in line with previous reports of phosphorylation-mediated regulation of Rcan1 by Gsk-3 β^55,56^. Although regulation in the reverse direction (of Gsk-3 β activity by Rcan1) was unexpected, we found decreased β-catenin levels in the aortas of *SM-Rcan1*^*−/−*^ and *EC-Rcan1*^*−/−*^ mice. Since Gsk-3 β-mediated phosphorylation rapidly triggers β-catenin proteolysis^46,47^, our results strongly suggest that Gsk-3 β activity is increased in the aortas of conditional *Rcan1*^*−/−*^ mice and therefore that the association of Rcan1 with Gsk-3 β inhibits its enzymatic activity in the aorta. β-catenin levels remained however unaffected in the aortas of constitutive *Rcan1*^*−/−*^ mice, suggesting that Gsk-3 β activity was not increased in these mice.

Another Gsk-3 β substrate is the RhoGTPase activator p190ARhoGAP^45^ whose phosphorylation by Gsk-3 β activates Rho in fibroblasts^44^. By using two independent Gsk-3 β inhibitors, we demonstrated that Gsk-3 β activity mediates not only basal MLC phosphorylation in aortic *Rcan1*^*fl/fl*^ SMCs but also the enhanced phosphorylation observed in aortic *Rcan1*^*−/−*^ SMCs. These results are in line with a recent study showing that lithium decreases MLC phosphorylation in ECs and attenuates endothelial permeability^57^. We propose a model in which Rcan1 inhibits Gsk-3 β-mediated phosphorylation of its other substrates; the loss of Gsk-3 β inhibition in conditional *Rcan1*^*−/−*^ cells would thus increase ROCK activation and therefore MLC phosphorylation and SMC contractility (Fig. 8h). In mice with this altered aortic wall structure, the sharp blood pressure rise induced by AngII would then increase vascular permeability and promote the accumulation of extravasated blood in the outer layers of the aorta tunica media (Fig. 8h).

Our study demonstrates that hypertensive treatment of conditional *Rcan1*^*−/−*^ mice recapitulates the main features of human IMH, a hypertension-associated disease that can either regress or progress to dissection in the short term or progress to aneurysm in the long term. We have uncovered a critical role for the RHO-ROCK-MLC axis in IMH formation and an unexpected contribution of GSK-3 β to this process. The mouse models developed here have great potential for research into the cellular and molecular mechanisms involved in IMH. Moreover, the prevention of aortic rupture and IMH formation upon blood pressure control and ROCK inhibition suggest potential therapeutic routes. Lithium has been used widely as a mood stabilizer since the mid-20^th^ century^58^. Lithium and other GSK-3 inhibitors have also reached clinical trials for diabetes and several types of cancer and neural diseases^59^. One recent clinical study showed that lithium decreases the risk of ischemic stroke in bipolar disorder patients^60^. Given that Gsk-3 β inhibition decreased MLC phosphorylation in our studies, it might be of interest to compare the incidence of IMH, aortic dissection, and aneurysm in the general population with that in a population of bipolar disorder patients with long-term exposure to lithium.

## Methods

### Mouse strains

*Myh11-Cre*^*ERT2*^ and *Cdh5-Cre*^*ERT2*^ mice express tamoxifen-inducible Cre specifically in SMCs and ECs, respectively, whereas *Ubc-Cre*^*ERT2*^ mice express tamoxifen-inducible Cre in a wide range of cells^34^. The Cre-reporter transgenic lines *Gt(ROSA)26Sor*^*tm9(CAG-tdTomato)Hze*^ (*Rosa26-LSL-Tomato)* and *Gt(ROSA)26Sor*^*tm1(EYFP)Cos*^ (*Rosa26-LSL-YFP)* were obtained from the Jackson Laboratory *(*JAX mice stock 007905 and 006148, respectively*)*. Constitutive *Rcan1* knockout mice (*Rcan1*^*−/−*^*)* were previously described^61^. Targeting vectors for conditional knockout of *Rcan1, Rcan1-1*, and *Rcan1-4* were engineered by flanking *Rcan1* exon 1, exon 4, or exon 6 with LoxP sites and introducing a *PGKgb2/neo* selection cassette flanked by Frt sites, as represented in Supplementary Fig. 1a. The *Rcan1*^fl/fl^ alleles were generated after crossing *Rcan1-1*( + /loxfrt), *Rcan1-4*( + /loxfrt), and *Rcan1*( + /loxfrt) mice with transgenic mice expressing Flp recombinase (JAX mice stock 003946). The *Cdh5-Cre*^*ERT2*^, *Myh11-Cre*^*ERT2*^, and *Ubc-Cre*^*ERT2*^ alleles were always used in hemizygosity. *Apoe*^−/−^ mice were obtained from Charles River (JAX mice stock 002052). All mouse strains were backcrossed with C57BL/6 J mice for more than nine generations. All mice were genotyped by PCR of tail samples using the primers indicated in Supplementary Table 2. For *Rcan1-1*^*fl/fl*^, *Rcan1-4*^*fl/fl*^, and *Rcan1*^*fl/fl*^ mice, the common primer “Cassette Neo common” was used before removal of the selection cassette. Cre recombination after tamoxifen treatment (delta band) was validated using the “Delta” primers indicated in Supplementary Table 2.

### Southern blotting

Genomic DNA was obtained from mESC clones previously electroporated with the transgene construction. Genomic digestion was performed with the specific restriction enzymes ApaI and BamHI. Digestion products were separated on a 0.7% agarose gel, transferred to a nylon membrane, and crosslinked with UV irradiation for 15 min. Membranes were incubated with previously radiolabeled ^32^P-probes and washed with SSC buffers in decreasing concentrations. Hybridization of the radiolabeled probe was detected with a STORM 840 radiography scanner (GE Healthcare).

### Animal procedures

Animal procedures were approved by the CNIC Ethics Committee and the Madrid regional authorities (Ref. PROEX 080/16) and conformed with EU Directive 2010/63EU and Recommendation 2007/526/EC regarding the protection of animals used for experimental and other scientific purposes, enforced in Spanish law under Real Decreto 1201/2005. For conditional gene deletion, mice received daily 1 mg i.p. injections of tamoxifen (Sigma Aldrich) on 5 consecutive days. Control animals (*Rcan1*^*+/+*^) were vehicle-treated littermates or tamoxifen-treated Cre-negative littermates. Constitutive *Rcan1*^*−/−*^ mice were also treated with tamoxifen as a control measure. AngII (dissolved in saline), amlodipine (dissolved in 50% DMSO), and noerpinephrine (dissolved in saline with 0.2% ascorbic acid), all from Sigma-Aldrich, were infused at 1 μg kg^−1^ min^−1^, 6 mg kg^−1^ day^−1^, and 34.5 mg kg^−1^ day^−1^, respectively, using subcutaneous osmotic minipumps (Alzet Corp). Fasudil (LC laboratories; 1 mg per ml), and a combination of hydralazine (Sigma-Aldrich; 320 µg per ml) plus hydrochlorothiazide (Sigma-Aldrich; 60 µg per ml) were administered in drinking water. For IMH and AAA experiments, aortas were dissected, perivascular tissue was carefully removed, and images of the aortas were taken with a Nikon SMZ800 scoop camera.

### Blood pressure measurements

Arterial blood pressure (BP) was measured by the mouse-tail cuff method using the automated BP-2000 Blood Pressure Analysis System (Visitech Systems, Apex, NC, USA). In brief, mice were trained for BP measurements every day for one week. After training, BP was measured one day before treatment to determine the baseline BP values in each mouse cohort. Measurements were repeated several times during experiments. BP measurements were recorded in mice located in a tail-cuff restrainer over a warmed surface (37 °C). Fifteen consecutive systolic and diastolic BP measurements were made, and the last 10 readings per mouse were recorded and averaged.

### In vivo ultrasound imaging

Images of the aorta were taken in isoflurane-anesthetized mice (2% isoflurane) by high-frequency ultrasound with a VEVO 2100 echography device (VisualSonics, Toronto, Canada) at 30-micron resolution. Maximal internal aortic diameters were measured at diastole using VEVO 2100 software, version 1.5.0. All recordings were made by a cardiologist or technician blinded to animal genotype and treatment. Measurements were taken before treatment initiation to determine the baseline diameters and were repeated several times during the experiment.

### In vivo permeability assays

Mice were immobilized in a bucket holder and a mixture of 1 mg ml^−1^ of each 10-kDa-rhodamine and 70 kDa-FITC labeled dextrans (Sigma-Aldrich) in saline were injected into the tail vein. Mice were euthanized after 20 min and perfused with PBS and 4% paraformaldehyde in PBS. Organs were collected and embedded in OCT for cryopreservation and sections were inspected by confocal microscopy.

### Cell procedures

To isolate and culture primary mouse vascular smooth muscle cells (vSMC), aortas were dissected and the adventitia was removed with forceps^21^. Tissue was digested with a solution of collagenase and elastase until a single-cell suspension was obtained. All experiments were performed during passages 3–7. vSMCs were infected over 5 h at a multiplicity of infection = 3. The medium was then replaced with fresh DMEM supplemented with 10% FBS, and cells were cultured for 3 more days for *Rcan1* genomic recombination analysis. When indicated, vSMC were treated for 1–2 h with 10 µM GSK-3β inhibitor VIII (Calbiochem; #361549), 50 µM LiCl, or 30 µM Fasudil (LC Laboratories). The HEK-293T (CRL-1573) and Jurkat (Clone E6-1, TIB-152) cell lines, required for high-titer lentivirus production and lentivirus titration, respectively, were purchased from ATCC. HEK293 is one of the cell lines listed in the database of commonly misidentified cell lines. However, the HEK-293T cell line used in this study was used only after receipt from ATCC or after resucitation from early stocks at low passage number. All cells were *mycoplasma*-negative.

For cell immunostaining, cells where fixed with 4% paraformaldehyde for 10 min and permeabilized with 0.3% Triton X-100 in PBS for 30 min. Samples were incubated overnight with Cy3-conjugated monoclonal anti-SMA (1:500, C6198, Sigma) and rabbit anti-p-MLC (1:50, #3671, Cell Signaling). Secondary antibody was AlexaFluor647-conjugated goat anti-rabbit (1:500; A-21245; BD Pharmingen). Images were acquired under a Leica SPE microscope with 40x or 63x oil immersion objective lenses and Leica LAS-AF acquisition software.

vSMC were incubated with Mitotracker Green (Thermo Fisher M7514; 500 nM) for 60 min in DMEM medium supplemented with 10% FBS. The total mitochondrial content was quantified by flow cytometry analysis of these cells. To determine the number of mitochondria per cell, confocal images stacks were captured with a Zeiss LSM 700 confocal microscope using a 63x oil immersion objective lens and ZEN acquisition software. The number of each object (mitochondrion) was quantified using the Imaris volume and surface plugging, which 3-D reconstructed mitochondria from from Z-stack images. Each experiment was done 4 times and 12–16 cells per condition were quantified.

### Lentivirus production and infection

The Cre coding sequence was obtained by PCR amplification and cloned into the pHRSIN lentiviral vector^62^. Pseudo-typed lentiviruses were produced by transient calcium phosphate transfection of HEK-293T cells and concentrated from culture supernatant by ultracentrifugation (2 h at 128,000xg; Ultraclear Tubes; SW28 rotor and Optima L-100 XP Ultracentrifuge; Beckman). Viruses were suspended in cold sterile PBS and titrated by transduction of Jurkat cells for 48 h. Transduction efficiency (GFP-expressing cells) and cell death (propidium iodide staining) were quantified by flow cytometry.

### Aortic histology

After sacrifice of mice by CO_2_ inhalation, aortas were perfused with saline, isolated, and fixed in 4% paraformaldehyde overnight at 4 °C. Paraffin cross sections (5 μm) from fixed aortas were stained with Masson’s trichrome (Masson), Hematoxylin and Eosin (HE), or Elastic Verhoeff–Van Gieson (EVG) or were used for immunofluorescence. Images were acquired under a Leica DM2500 microscope with 20×, 40×, or 63xHCX PL Fluotar objective lenses and Leica Application Suite V3.5.0 acquisition software. IMH were considered as contained hematomas featuring bleeding within the medial layer in the absence of intimal tear or flap formation. The area of hemorrhage was quantified using ImageJ software, splitting the channels and applying the same threshold in all samples to highlight the blood accumulation. Hematomas were considered as individual events, and when an animal had more than one hematoma, all of them where quantified and included in the analysis when possible. For immunofluorescence, deparaffinized sections were rehydrated, boiled to retrieve antigens (10 mM citrate buffer, pH6), and blocked for 45 min with 10% goat serum plus 2% BSA in PBS. Samples were incubated with the following antibodies for immunofluorescence: Cy3-conjugated monoclonal anti-SMA (1:500, C6198, Sigma), rabbit anti-cd31 (1:50, ab28364, Abcam), and rabbit anti-p-MLC (1:50, #3671, Cell Signaling). For permeability assays, cryosections (8 µm) were obtained, blocked with 10% goat serum plus 2% BSA in PBS for 1 h, and then incubated with hamster anti-cd31 (1:200; MAB1398Z; Millipore) and rabbit anti-p-MLC primary antibodies. Specificity was determined by substituting the primary antibody with unrelated IgG (diluted as antigen-specific antibodies; sc-2025, Santa Cruz). For immunofluorescence, secondary antibodies were AlexaFluor647-conjugated goat anti-hamster (1:500, 127–605–160, Jackson Inmuno Reasearch) and AlexaFluor647-conjugated goat anti-rabbit (1:500; A-21245, BD Pharmingen). Sections were mounted with DAPI in Citifluor AF4 mounting medium (Aname). For Apoptosis staining, aortas were analyzed with the ApopTag TdT enzyme kit (S7165, Millipore). Images were acquired with a Nikon A1R confocal microscope fitted with a 20x air objective or a 40x or 63x oil immersion objective and using Nikon NIS-Elements software (1024 × 1024 pixels, 8bits) or with a Zeiss LSM 700 confocal microscope fitted with a 20x air objective or a 40x or 63x oil immersion objective and using Zeiss ZEN software (2448 × 2448 pixels, 8 bits).

### Aortic whole-mount immunostaining

Aortas from euthanized mice were isolated after perfusion with saline followed by 4% paraformaldehyde. Aortas were then longitudinally cut and pinned to a gelatin-coated plate and fixed in 4% paraformaldehyde overnight at 4 °C. The tissue was permeabilized and blocked with 0.3% Triton X-100, 10% goat serum, 5% BSA, and FcBlock (1:100; Rat Anti-Mouse CD16/CD32; BD Bioscience) in PBS for 1 h. Aortas were incubated overnight at room temperature hamster anti-cd31 (1:200; MAB1398Z; Millipore) in blocking buffer. After abundant washes with PBS, samples were incubated overnight with AlexaFluor647-conjugated goat anti-hamster secondary antibody (1:500, 127-605-160, Jackson Inmuno Reasearch) and DAPI (1:10,000). Aortas were mounted in Citifluor AF4 mounting medium (Aname). Images were acquired with a Nikon A1R confocal microscope fitted with a 20x air objective or a 40x oil immersion objective and using Nikon NIS-Elements software (1024 × 1024 pixels, 8bits).

### Transmission electron microscopy

Male mice were euthanized and transcardially perfused with saline followed by fixation solution (50 ml 4% PFA in PBS). Dissected aortas were additionally fixed in 3% glutaraldehyde for 24hs and embedded in resin (Durcupan ACM Fluka, Sigma-Aldrich) for transverse sectioning. Ultrathin sections were cut and counterstained at the Electron Microscopy facility (SIdI service) at the Universidad Autónoma de Madrid. Sections were imaged with a transmission electron microscope (Jeol Jem 1010, 80 kV, Jeol Ltd. Tokyo, Japan) and recorded with a Gatan camera (Orius, SC200W, Pleasenton, California) at the indicated magnifications.

### Immunoprecipitation and mass spectrometry

For immunoprecipitation, protein lysates from mouse brains (5 mg) or from cultured vSMCs (1 mg) were prepared in lysis buffer (50 mM HEPES pH 7.9, 150 mM NaCl, 1% NP-40, 5% glycerol, 1% benzonase, 1 mM MgCl2), precleared with 50 μl of underivatized agarose beads (Co-IP, Pierce) for 1 h at 4 °C, and then incubated overnight with 20 µl sepharose beads conjugated to anti-Rcan1 antibody (Sigma) using the Pierce Co-IP kit (ThermoFisher). Beads were washed three times in lysis buffer and two times with lysis buffer without detergent. Immunoprecipitation efficiency was analyzed by immunoblot with anti-Rcan1 antibody. Immune complexes from brain lysates were trypsin digested followed by nanoliquid chromatography coupled to mass spectrometry for protein identification and quantification by spectral counting^63^. Peptide were identified from MS/MS data by the probability ratio method^64^. False discovery rates (FDR) of peptide identifications were calculated using the refined method^65,66^; 1%FDR was used as the criterion for peptide identification. Raw mass spectrometer output data are available at PeptideAtlas [http://www.peptideatlas.org/PASS/PASS01228]. Aortic protein lysates and immunoprecipitated complexes were also analyzed by immunobloting with anti-GSK3β antibody.

### Immunoblotting

To prepare aortic protein lysates, mouse aortic samples were isolated, frozen in liquid nitrogen, and then homogenized using a mortar and an automatic bead homogenizer (MagNA lyzer, Roche). Protein extracts were obtained in ice-cold RIPA buffer (50 mM NaCl, 50 mM Tris HCl pH8, 1% NP40, 0.1% SDS, 0.5% sodium deoxycholate) supplemented with protease, phosphatase, and kinase inhibitors. Cultured cells were washed with ice-cold PBS and lysed in RIPA buffer.

Immunoprecipitated proteins and protein lysates were separated under reducing conditions on SDS-polyacrylamide gels and transferred to nitrocellulose membranes. Proteins were detected with the following primary antibodies: rabbit anti-Rcan1 (1:1000; D6694, Sigma), anti-p-MLC (1:1000; #3671, Cell Signaling), mouse monoclonal anti-β-catenin (1:1000; 610153, BD Biosciences), mouse monoclonal anti-GSK3β (1:1000; 610202, BD Biosciences), mouse monoclonal anti-alpha tubulin (1:40,000; T 6074 Sigma-Aldrich), and anti-GAPDH (1:5000; ab8245 Abcam). HRP-conjugated secondary antibodies were detected with enhanced chemiluminescence (ECL) detection reagent (Millipore).

### RT and quantitative PCR

Aortas were extracted after perfusion with 5 ml saline solution, and the adventitia layer was discarded. Frozen tissue was homogenized using a mortar and an automatic bead homogenizer (MagNA Lyzer). Total RNA was isolated with TRIZOL (Life Technologies). Total RNA (2 μg) was reverse transcribed at 37 °C for 50 min in a 20 µl reaction mix containing 200U Moloney murine leukemia virus (MMLV) reverse transcriptase (Life Technologies), 100 ng random primers, and 40U RNase Inhibitor (Life Technologies). Real-time quantitative RT-PCR was performed with the RT-PCR primers indicated in Supplementary Table 2.

RT-qPCR reactions were performed in triplicate with SYBR-master mix (Applied Biosystems). Probe specificity was checked by post-amplification melting-curve analysis; for each reaction, only one Tm peak was produced. The amount of target mRNA in samples was estimated by the 2^−∆CT^ relative quantification method, using *Gapdh* or *Hprt* for normalization. Fold ratios were calculated relative to control animals.

### Statistical analysis

GraphPad Prism software 7.0 was used for the analysis. Data normality and homoscedasticity were assessed by Shapiro-Wilk and Bartlett’s tests, respectively. Appropriate tests were chosen according to the data distribution. For survival curves, differences were analyzed with the Log-rank (Mantel-Cox) test. Incidence of IMH or AAA (presence of at least one event per animal) was represented as percentage of affected animals. Statistics were analyzed by the Chi-square test for trend. The aortic diameter data are presented as box and whiskers plots; bars represent maximal and minimal values. Blood pressure, fluorescence intensity, and RT-PCR data were compared to the values in wt mice or to baseline measurements, as appropriate. Gaussian data were analyzed by one-way or two-way ANOVA and the Bonferroni post-hoc test or Tukey post-hoc test (experiments with ≥ 3 groups), as appropriate. Non-gaussian data were compared with Kruskal-Wallis with Dunn’s multiple comparison post-hoc test. Statistical significance was assigned at *p < 0.05, **p < 0.01, ***p < 0.001, and ****p < 0.0001.

The numbers of animals used are indicated in the corresponding figure legends. Sample size was chosen empirically according to our experience in the calculation of experimental variability; no statistical method was used to predetermine sample size, and no data were excluded. All experiments were carried out with at least three biological replicates. Experimental groups were balanced in terms of animal age, and weight. Only male mice were used because the *Myh11-Cre*^*ERT2*^ transgene was inserted in the Y chromosome (https://www.jax.org/strain/019079). No randomization was used to allocate animals to experimental groups and investigators were not blinded to group allocation during experiments or to outcome assessments. Animals were genotyped before experiments, caged together (regardless of their genotype), and treated in the same way.

## Electronic supplementary material

Supplementary Information

## Data Availability

Proteomics data are available at PeptideAtlas under the accession code PASS01228. Other datasets generated and/or analyzed during the current study are available form the corresponding authors on reasonable request.

## References

[1] Evangelista A (2003). Long-term follow-up of aortic intramural hematoma: predictors of outcome. Circulation.

[2] Alomari IB (2014). Aortic intramural hematoma and its complications. Circulation.

[3] Evangelista A (2004). Aortic intramural haematoma: remarks and conclusions. Heart.

[4] Song JK (2014). Update in acute aortic syndrome: intramural hematoma and incomplete dissection as new disease entities. J. Cardiol..

[5] Humphrey JD, Schwartz MA, Tellides G, Milewicz DM (2015). Role of mechanotransduction in vascular biology: focus on thoracic aortic aneurysms and dissections. Circ. Res..

[6] Milewicz DM, Prakash SK, Ramirez F (2017). Therapeutics targeting drivers of thoracic aortic aneurysms and acute aortic dissections: insights from predisposing genes and mouse models. Annu. Rev. Med..

[7] Nienaber CA (2016). Aortic dissection. Nat. Rev. Dis. Prim..

[8] Hagan PG (2000). The International Registry of Acute Aortic Dissection (IRAD): new insights into an old disease. JAMA.

[9] Landenhed M (2015). Risk profiles for aortic dissection and ruptured or surgically treated aneurysms: a prospective cohort study. J. Am. Heart Assoc..

[10] Rateri DL (2014). Angiotensin II induces region-specific medial disruption during evolution of ascending aortic aneurysms. Am. J. Pathol..

[11] Daugherty A, Manning MW, Cassis LA (2000). Angiotensin II promotes atherosclerotic lesions and aneurysms in apolipoprotein E-deficient mice. J. Clin. Invest..

[12] Kanematsu Y (2010). Pharmacologically induced thoracic and abdominal aortic aneurysms in mice. Hypertension.

[13] Weintraub NL (2009). Understanding abdominal aortic aneurysm. N. Engl. J. Med..

[14] Esteban V (2011). Regulator of calcineurin 1 mediates pathological vascular wall remodeling. J. Exp. Med..

[15] Davies KJ (2007). Renaming the DSCR1/Adapt78 gene family as RCAN: regulators of calcineurin. FASEB J..

[16] Fuentes JJ, Pritchard MA, Estivill X (1997). Genomic organization, alternative splicing, and expression patterns of the DSCR1 (Down syndrome candidate region 1) gene. Genomics.

[17] Cano E, Canellada A, Minami T, Iglesias T, Redondo JM (2005). Depolarization of neural cells induces transcription of the Down syndrome critical region 1 isoform 4 via a calcineurin/nuclear factor of activated T cells-dependent pathway. J. Biol. Chem..

[18] Crawford DR (1997). Hamster adapt78 mRNA is a Down syndrome critical region homologue that is inducible by oxidative stress. Arch. Biochem. Biophys..

[19] Ermak G, Davies KJ (2002). Gene expression in Alzheimer’s disease. Drugs Today (Barc).

[20] Minami T (2004). Vascular endothelial growth factor- and thrombin-induced termination factor, Down syndrome critical region-1, attenuates endothelial cell proliferation and angiogenesis. J. Biol. Chem..

[21] Oller J (2015). C/EBPbeta and nuclear factor of activated T cells differentially regulate Adamts-1 induction by stimuli associated with vascular remodeling. Mol. Cell. Biol..

[22] Wang Y (2002). Direct biomechanical induction of endogenous calcineurin inhibitor Down syndrome critical region-1 in cardiac myocytes. Am. J. Physiol. Heart Circ. Physiol..

[23] Yang J (2000). Independent signals control expression of the calcineurin inhibitory proteins MCIP1 and MCIP2 in striated muscles. Circ. Res..

[24] Baek KH (2009). Down’s syndrome suppression of tumour growth and the role of the calcineurin inhibitor DSCR1. Nature.

[25] Hoeffer CA (2007). The Down syndrome critical region protein RCAN1 regulates long-term potentiation and memory via inhibition of phosphatase signaling. J. Neurosci..

[26] Mendez-Barbero N (2013). A major role for RCAN1 in atherosclerosis progression. EMBO Mol. Med..

[27] Ryeom S (2008). Targeted deletion of the calcineurin inhibitor DSCR1 suppresses tumor growth. Cancer Cell..

[28] Yang YJ (2009). Rcan1 negatively regulates Fc epsilonRI-mediated signaling and mast cell function. J. Exp. Med..

[29] Rothermel B (2000). A protein encoded within the Down syndrome critical region is enriched in striated muscles and inhibits calcineurin signaling. J. Biol. Chem..

[30] Kingsbury TJ, Cunningham KW (2000). A conserved family of calcineurin regulators. Genes Dev..

[31] Vega RB (2003). Dual roles of modulatory calcineurin-interacting protein 1 in cardiac hypertrophy. Proc. Natl. Acad. Sci. USA.

[32] Wang Y (2010). Ephrin-B2 controls VEGF-induced angiogenesis and lymphangiogenesis. Nature.

[33] Wirth A (2008). G12-G13-LARG-mediated signaling in vascular smooth muscle is required for salt-induced hypertension. Nat. Med..

[34] Ruzankina Y (2007). Deletion of the developmentally essential gene ATR in adult mice leads to age-related phenotypes and stem cell loss. Cell. Stem. Cell..

[35] Hall ME, Smith G, Hall JE, Stec DE (2011). Systolic dysfunction in cardiac-specific ligand-inducible MerCreMer transgenic mice. Am. J. Physiol. Heart Circ. Physiol..

[36] Parra V (2018). Down syndrome critical region 1 Gene, Rcan1, helps maintain a more fused mitochondrial network. Circ. Res..

[37] Nakayama H, Nishida K, Otsu K (2016). Macromolecular degradation systems and cardiovascular aging. Circ. Res..

[38] Tan WSD, Liao W, Zhou S, Mei D, Wong WF (2018). Targeting the renin-angiotensin system as novel therapeutic strategy for pulmonary diseases. Curr. Opin. Pharmacol..

[39] van Thiel BS, van der Pluijm I, te Riet L, Essers J, Danser AH (2015). The renin-angiotensin system and its involvement in vascular disease. Eur. J. Pharmacol..

[40] Julius S (1988). Amlodipine in hypertension: an overview of the clinical dossier. J. Cardiovasc. Pharmacol..

[41] Zhang Y, Moreland S, Moreland RS (1994). Regulation of vascular smooth muscle contraction: myosin light chain phosphorylation dependent and independent pathways. Can. J. Physiol. Pharmacol..

[42] Kaneko-Kawano T (2012). Dynamic regulation of myosin light chain phosphorylation by Rho-kinase. PLoS One.

[43] Liao JK, Seto M, Noma K (2007). Rho kinase (ROCK) inhibitors. J. Cardiovasc. Pharmacol..

[44] Jiang W (2008). p190A RhoGAP is a glycogen synthase kinase-3-beta substrate required for polarized cell migration. J. Biol. Chem..

[45] Sun T, Rodriguez M, Kim L (2009). Glycogen synthase kinase 3 in the world of cell migration. Dev. Growth Differ..

[46] McCubrey JA (2014). Multifaceted roles of GSK-3 and Wnt/beta-catenin in hematopoiesis and leukemogenesis: opportunities for therapeutic intervention. Leukemia.

[47] van Kappel EC, Maurice MM (2017). Molecular regulation and pharmacological targeting of the beta-catenin destruction complex. Br. J. Pharmacol..

[48] Freland L, Beaulieu JM (2012). Inhibition of GSK3 by lithium, from single molecules to signaling networks. Front. Mol. Neurosci..

[49] Conde S, Perez DI, Martinez A, Perez C, Moreno FJ (2003). Thienyl and phenyl alpha-halomethyl ketones: new inhibitors of glycogen synthase kinase (GSK-3beta) from a library of compound searching. J. Med. Chem..

[50] Langheinrich AC (2006). Correlation of vasa vasorum neovascularization and plaque progression in aortas of apolipoprotein E(-/-)/low-density lipoprotein(-/-) double knockout mice. Arterioscler. Thromb. Vasc. Biol..

[51] Ballesteros-Martinez C (2017). Endothelial regulator of calcineurin 1 promotes barrier integrity and modulates histamine-induced barrier dysfunction in anaphylaxis. Front. Immunol..

[52] Cassis LA (2009). ANG II infusion promotes abdominal aortic aneurysms independent of increased blood pressure in hypercholesterolemic mice. Am. J. Physiol. Heart Circ. Physiol..

[53] Burgoyne JR, Eaton P (2010). Oxidant sensing by protein kinases a and g enables integration of cell redox state with phosphoregulation. Sensors (Basel).

[54] Dasgupta SK, Le A, Vijayan KV, Thiagarajan P (2017). Dasatinib inhibits actin fiber reorganization and promotes endothelial cell permeability through RhoA-ROCK pathway. Cancer Med..

[55] Jung MS (2011). Regulation of RCAN1 protein activity by Dyrk1A protein-mediated phosphorylation. J. Biol. Chem..

[56] Vega RB, Yang J, Rothermel BA, Bassel-Duby R, Williams RS (2002). Multiple domains of MCIP1 contribute to inhibition of calcineurin activity. J. Biol. Chem..

[57] Bosche B (2016). Low-dose lithium stabilizes human endothelial barrier by decreasing MLC phosphorylation and universally augments cholinergic vasorelaxation capacity in a direct manner. Front. Physiol..

[58] Shorter E (2009). The history of lithium therapy. Bipolar Disord..

[59] Pandey MK, DeGrado TR (2016). Glycogen synthase kinase-3 (GSK-3)-targeted therapy and imaging. Theranostics.

[60] Lan CC (2015). A reduced risk of stroke with lithium exposure in bipolar disorder: a population-based retrospective cohort study. Bipolar Disord..

[61] Porta S (2007). RCAN1 (DSCR1) increases neuronal susceptibility to oxidative stress: a potential pathogenic process in neurodegeneration. Hum. Mol. Genet..

[62] Escolano A (2014). Specific calcineurin targeting in macrophages confers resistance to inflammation via MKP-1 and p38. EMBO J..

[63] Villarroya-Beltri C (2013). Sumoylated hnRNPA2B1 controls the sorting of miRNAs into exosomes through binding to specific motifs. Nat. Commun..

[64] Martinez-Bartolome S (2008). Properties of average score distributions of SEQUEST: the probability ratio method. Mol. Cell. Proteom..

[65] Bonzon-Kulichenko E, Garcia-Marques F, Trevisan-Herraz M, Vazquez J (2015). Revisiting peptide identification by high-accuracy mass spectrometry: problems associated with the use of narrow mass precursor windows. J. Proteome Res..

[66] Navarro P, Vazquez J (2009). A refined method to calculate false discovery rates for peptide identification using decoy databases. J. Proteome Res..

