# Supplementary material for: Conditional deletion of Rcan1 predisposes to hypertension-mediated intramural hematoma and subsequent aneurysm and aortic rupture
Source: Nat Commun. 2018 Nov 15;9:4795. doi: 10.1038/s41467-018-07071-7 (PMC6237779; doi:10.1038/s41467-018-07071-7)
Supplement: Supplementary file 1 — Supplementary Information [file 41467_2018_7071_MOESM1_ESM.pdf]

SUPPLEMENTARY INFORMATION

Conditional deletion of *Rcan1* in vascular cells predisposes to hypertension-mediated aortic rupture and intramural hematoma

Villahoz *et al.*

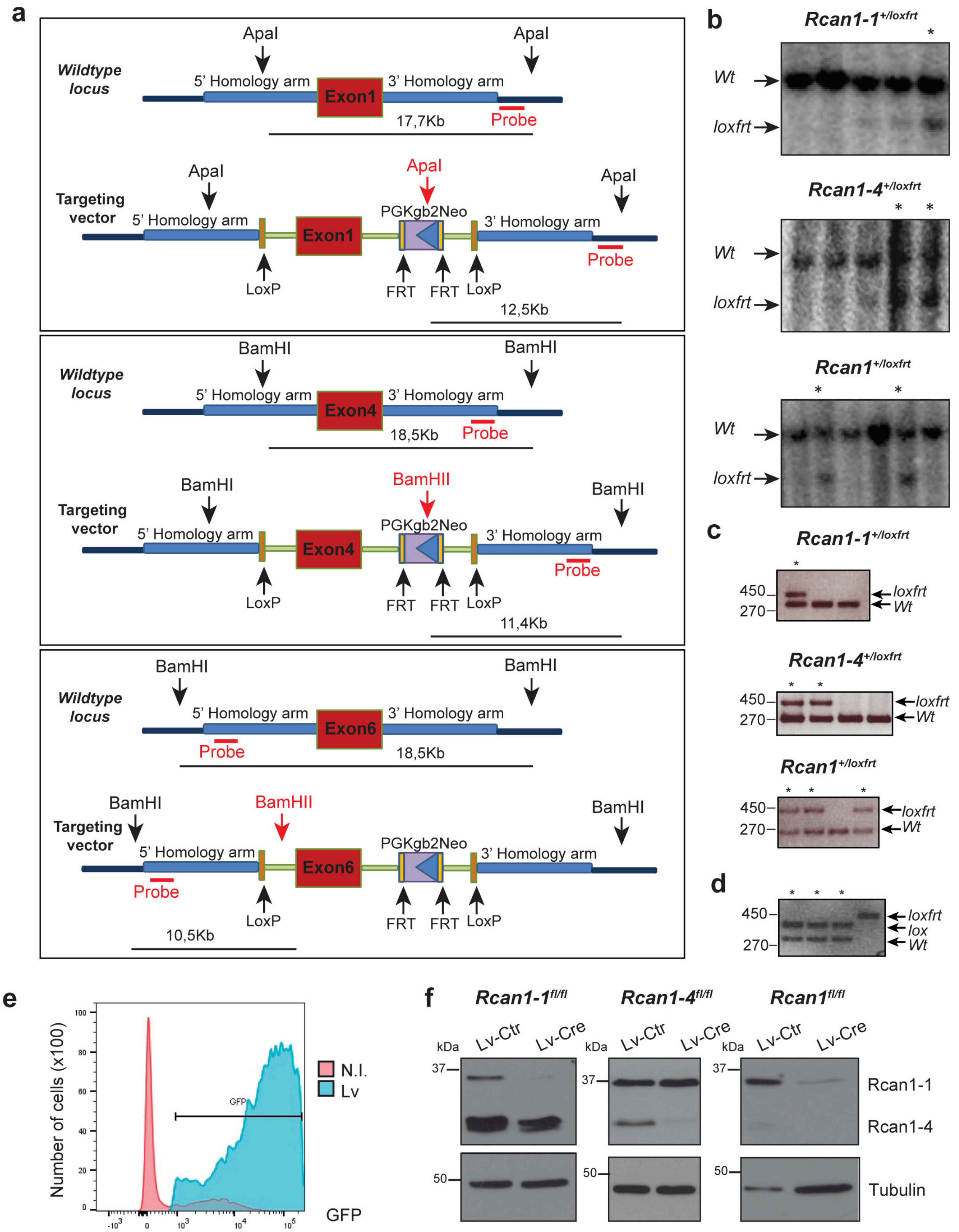

Supplementary Figure 1

Supplementary Figure 1. Targeting strategy for conditional deletion of *Rcan1-1*, *Rcan1-4,* or both isoforms. (a) Schematic representation of the wild-type *Rcan1* genomic regions encompassing exon 1, exon 4, and exon 6, together with the corresponding targeting vectors. The schemes indicate the approximate location of LoxP and Frt sites, the *PGKgb2/Neo* cassette, the restriction enzyme sites, and the probes used for screening of correctly recombined clones. **(b)** Southern blot analysis of mESC clones electroporated with targeting vectors specific for exon 1 (top panel), exon 4 (middle panel), or exon 6 (bottom panel). Asterisks denote correctly recombined clones (loxfrt allele). **(c)** PCR analysis of genomic DNA from the first generation of chimera progenies. *Rcan1*$^{+/loxfrt}$ indicates PCR products specific for positively recombined alleles. Asterisks indicate lanes from animals positive for germline transmission. **(d)** PCR of genomic DNA from the first generation of mice bred with FLP-expressing mice to remove the *PGKgb2/Neo* cassette. Asterisks mark lanes with DNA from recombined mice. The position of PCR products specific for the wt, non-recombined control (Neo-LoxP), and recombined (loxP) alleles are indicated. **(e)** Flow cytometry analysis of GFP expression in vSMCs transduced with lentiviral vectors encoding GFP (Lv). N.I., non-infected. **(f)** Representative Rcan1 and tubulin (loading control) immunoblot analysis of vSMCs transduced with Cre-encoding (Lv-Cre) or control (Lv-Ctr) lentiviral vectors (n=3 per genotype).

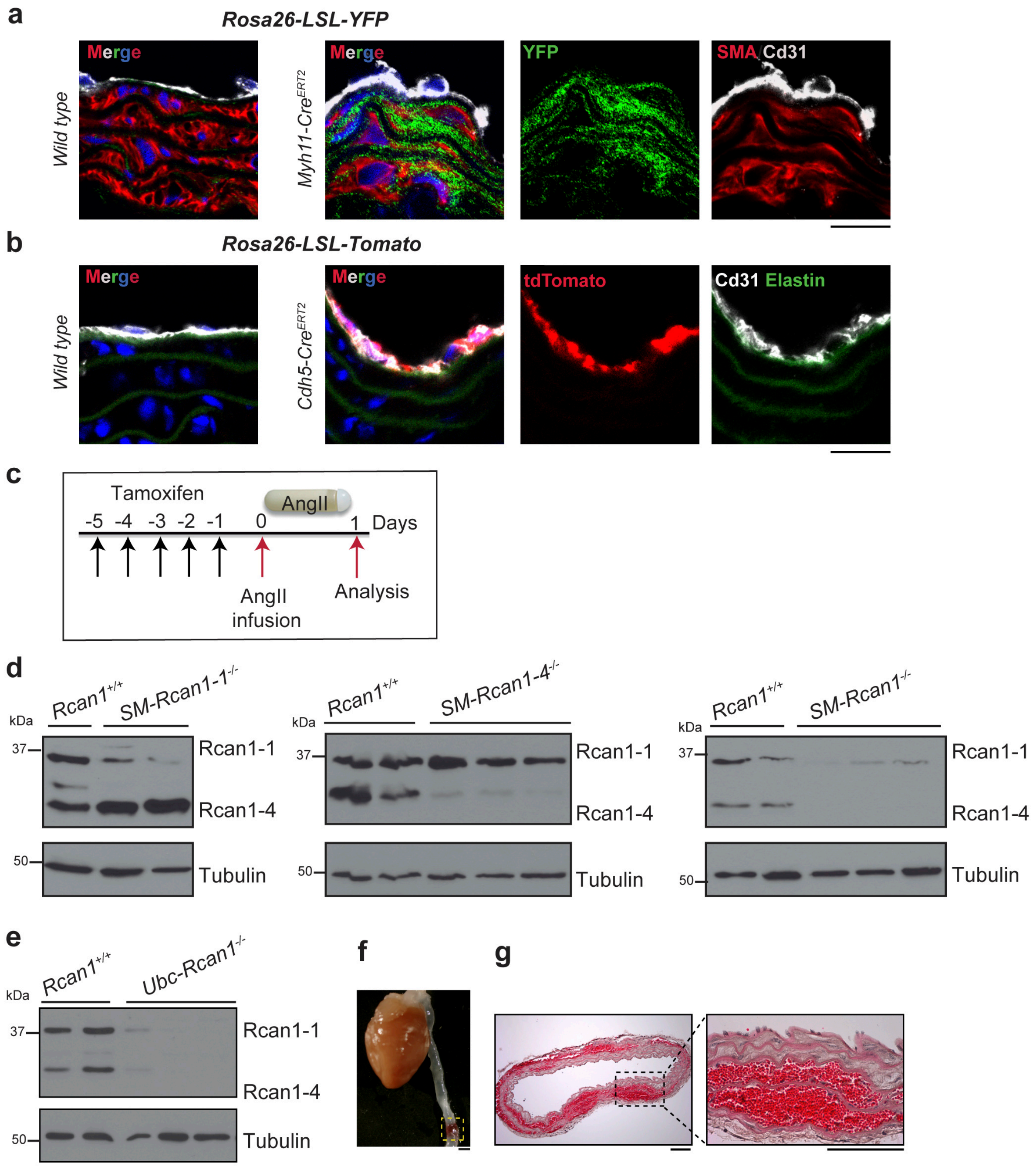

Supplementary Figure 2

Supplementary Figure 2. Inducible and tissue specific Cre/loxP-mediated recombination in the *Rcan1* locus. (a) Representative images of YFP (green) and DAPI (blue) fluorescence and SMA (red) and Cd31 (pale gray) immunofluorescence on aortic sections from 8-week-old wt and tamoxifen-treated *Myh11-Cre^{ERT2};Rosa26-LSL-YFP* mice (n=4 of each genotype). Scale bar, 20µm. **(b)** Representative images of tdTomato (red), elastin (green), and DAPI (blue) fluorescence and Cd31 immunofluorescence (pale gray) on aortic sections from 8-week-old wt and tamoxifen-treated *Cdh5-Cre^{ERT2};Rosa26-LSL-Tomato* mice (n=4 of each genotype). Scale bar, 20µm. **(c)** Experimental design: 8-week-old *SM-Rcan1-1$^{fl/fl}$*, *SM-Rcan1-4$^{fl/fl}$*, *SM-Rcan1$^{fl/fl}$*, and *Ubc-Rcan1$^{fl/fl}$* mice were injected with tamoxifen on 5 consecutive days before AngII minipump implantation. Aortae were extracted from euthanized mice 24h after minipump implantation. Representative Rcan1 and tubulin (loading control) immunoblot analysis of total aortic extracts from **(d)** *Rcan1$^{+/+}$*, *SM-Rcan1-1$^{-/-}$*, *SM-Rcan1-4$^{-/-}$*, and *SM-Rcan1$^{-/-}$* mice (n=3 per genotype) and **(e)** *Rcan1$^{+/+}$* and *Ubc-Rcan1$^{-/-}$* mice (n=3 per genotype). **(f)** Representative image of an aortic lesion in *SM-Rcan1$^{-/-}$* mice treated with AngII for 24h and **(g)** representative images of hematoxylin-eosin staining on an aortic section from the lesion area in (f) (n=3). Scale bars, 1mm (f); 100µm (g).

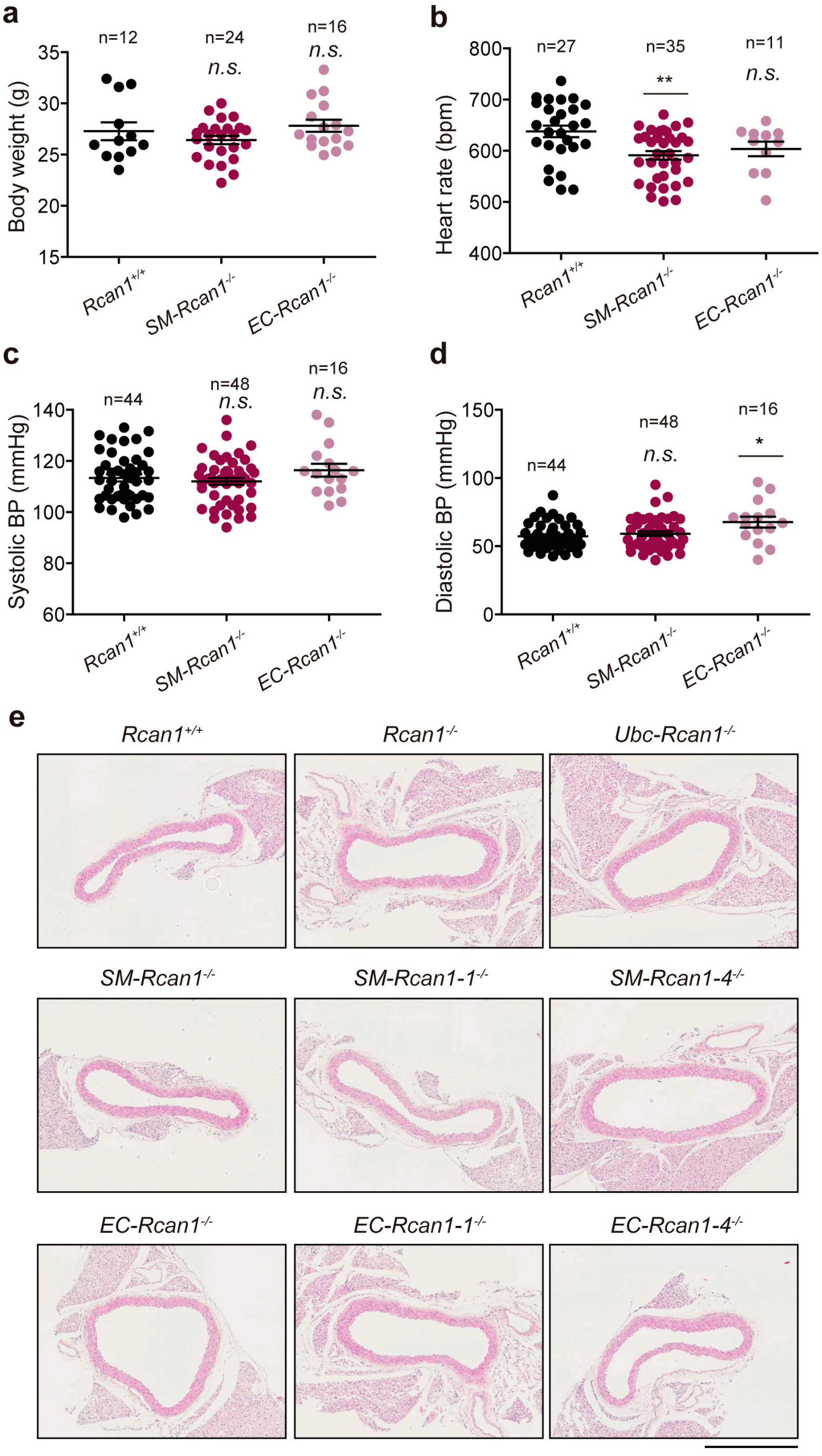

Supplementary Figure 3

Supplementary Figure 3. Conditional *Rcan1* deletion causes no evident symptoms in the absence of AngII. (a) Body weight, **(b)** heart rate, **(c)** systolic blood pressure (BP), and **(d)** diastolic BP. Each data point denotes an individual mouse, and the horizontal bars denote the mean (long bar) and the s.e.m. The number of mice per group is indicated in each panel. One-way ANOVA with Tukey multiple comparison post-hoc test (b and c) and Kruskal-Wallis with Dunn multiple comparison post-hoc test (a and d); *p<0.05, **p<0.01 vs *Rcan1*$^{+/+}$; *n.s.*, non-significant vs *Rcan1*$^{+/+}$. **(e)** Representative images of hematoxylin-eosin staining on aortic sections from the indicated mice. Scale bar, 500μm.

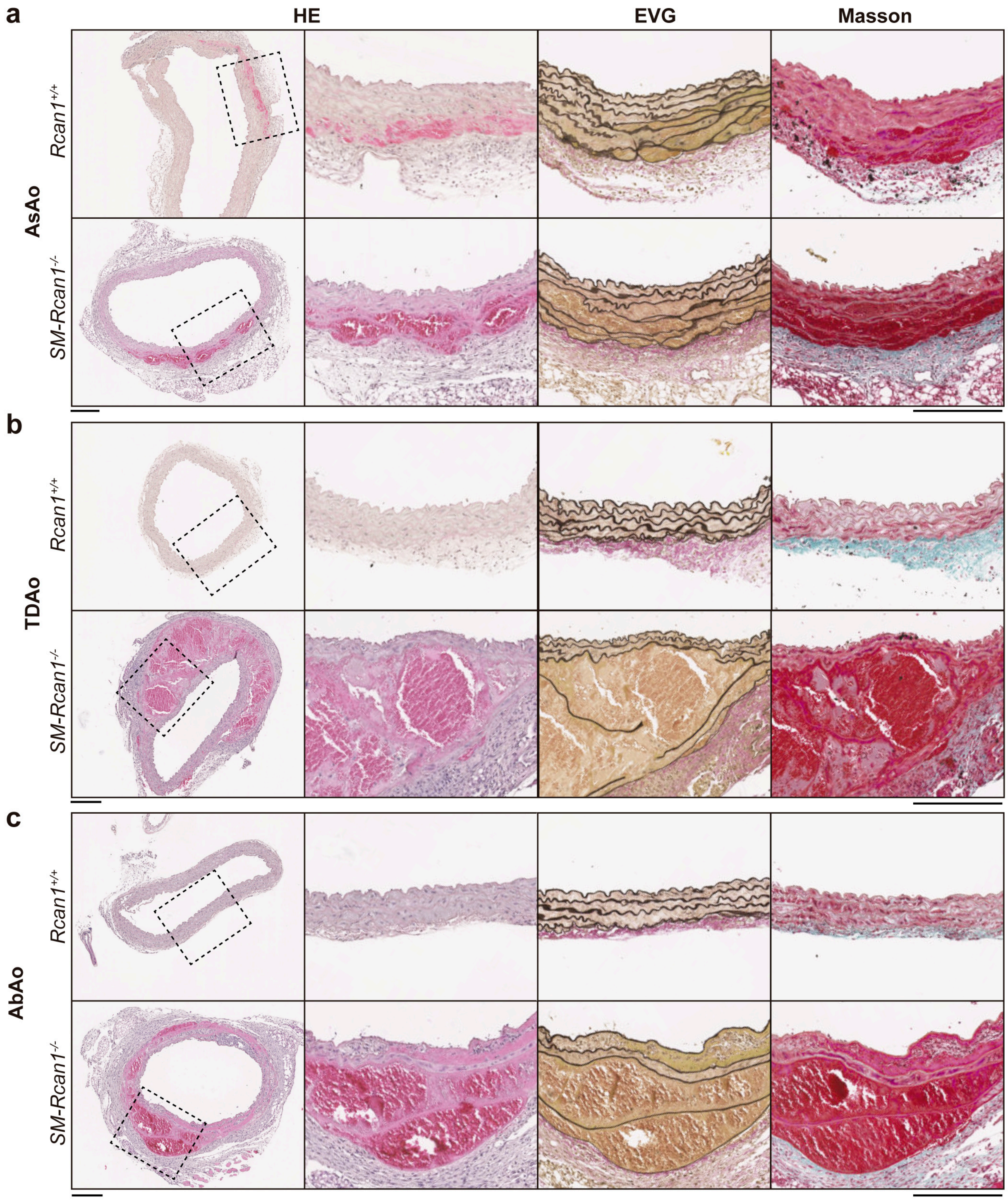

Supplementary Figure 4. Histological characterization of aortic IMH. Images show Hematoxylin-eosin (HE), Masson's trichrome (Masson), and Elastic Van Gieson (EVG) staining on AsAo **(a),** TDAo **(b),** and AbAo **(c)** sections from *Rcan1*$^{+/+}$ and *SM-Rcan1*$^{-/-}$ mice. Scale bars, 200μm.

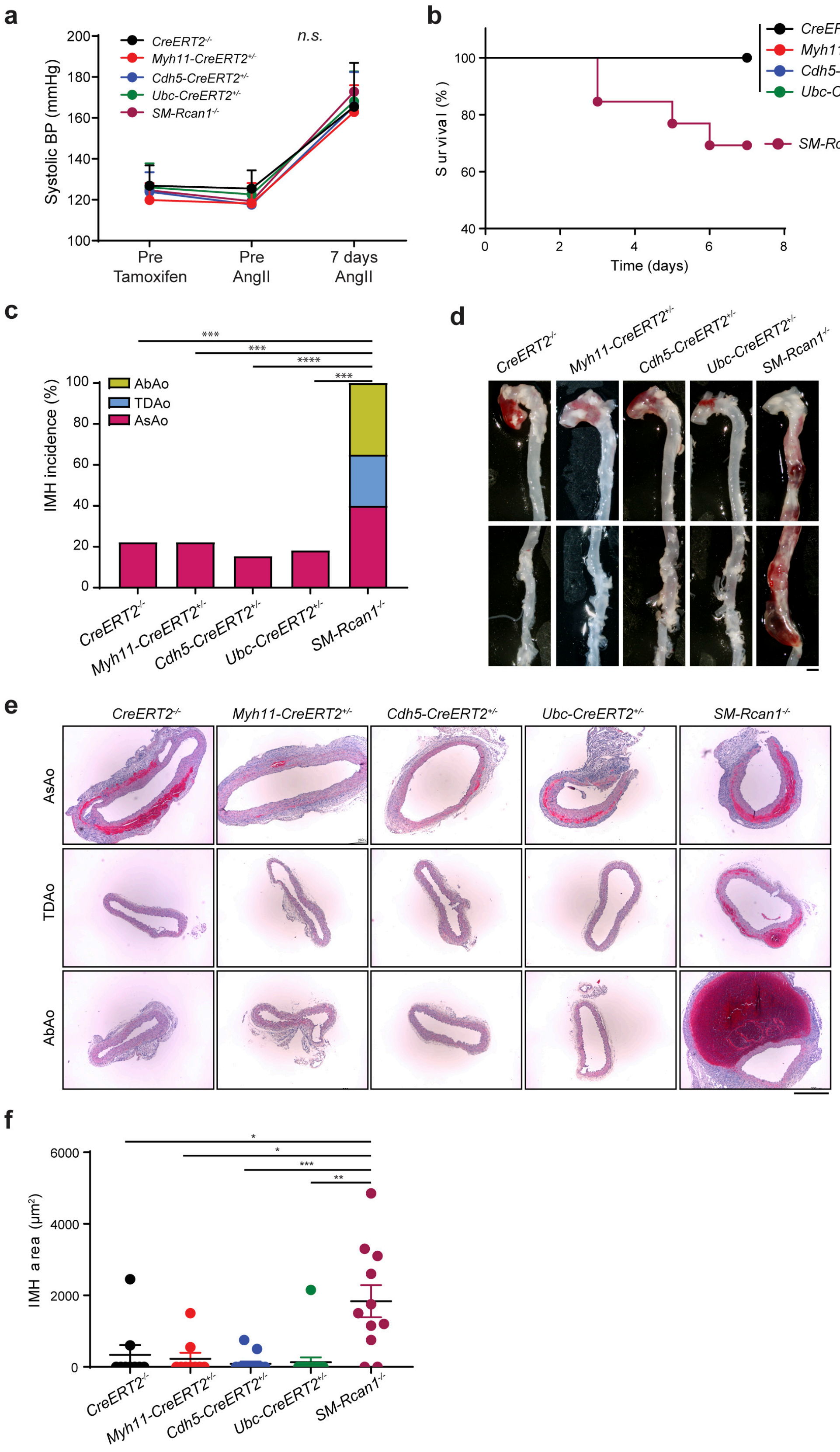

Supplementary Figure 5

Supplementary Figure 5. Cre activation in the aorta of *Rcan1*$^{+/+}$ mice does not predispose to IMH or aortic rupture. Eight-week-old male mice were treated with tamoxifen for 5 consecutive days before the implantation of osmotic minipumps for AngII infusion (1 μg/kg/min). **(a)** Systolic BP values of Cre-negative *Rcan1*$^{fl/fl}$ (*CreERT2*$^{-/-}$; n=9), *Rcan1*$^{+/+}$*;Myh11-Cre*$^{ERT2+/-}$ (*Myh11-CreERT2*$^{+/-}$; n=9), *Rcan1*$^{+/+}$*;Cdh5-Cre*$^{ERT2+/-}$ (*Cdh5-CreERT2*$^{+/-}$; n=13), *Rcan1*$^{+/+}$*;Ubc-Cre*$^{ERT2+/-}$ (*Ubc-CreERT2*$^{+/-}$; n=11), and *SM-Rcan1*$^{-/-}$ (n=10) mice immediately before the initiation of tamoxifen treatment (Pre tamoxifen) and AngII infusion (Pre Ang-II), and at the end of the experiment (7 days Ang-II). Kruskal-Wallis with Dunn multiple comparison post-hoc test, *n.s.*, non-significant among genotypes. **(b)** Survival curves of these mice after AngII osmotic minipump implantation. Log-rank (Mantel-Cox) test, *p<0.05 vs *SM-Rcan1*$^{-/-}$. All deaths were due to aortic rupture. **(c)** End-of-experiment IMH incidence in the AsAo, TDAo, and AbAo. Chi-square distribution, ****p<0.0001; ***p<0.001 vs *SM-Rcan1*$^{-/-}$. **(d)** Representative images of macroscopic hematomas in aortas from mice euthanized at the end of the experiment. Scale bar, 1mm. **(e)** Representative images of aortic sections from the same cohorts of mice stained with hematoxylin-eosin. Scale bar, 500μm. **(f)** Hemorrhage area in aortic sections from these mice. Kruskal-Wallis with Dunn multiple comparison post-hoc test, ***p<0.001, **p<0.01, *p<0.05 vs *SM-Rcan1*$^{-/-}$. Each data point denotes an individual mouse, and the horizontal bars denote the mean (long bar) and the s.e.m.

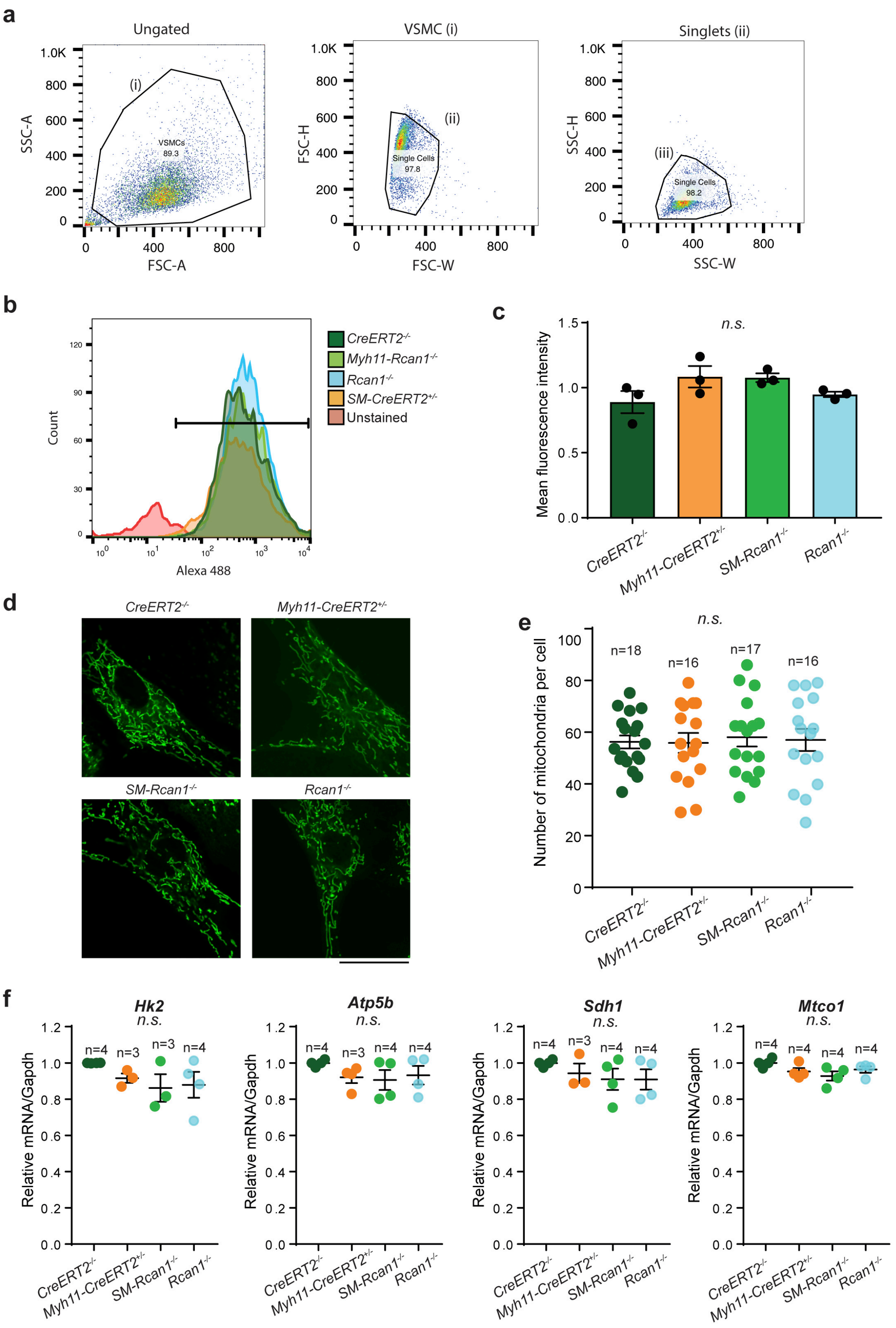

Supplementary Figure 6

Supplementary Figure 6. Absence of mitochondrial aberrations upon Cre activation or inducible *Rcan1* deletion. (**a**) Gating strategy in flow cytometry studies with vSMCs stained with Mitotracker. Cells were initially gated based on size and granularity using forward scatter area (FSC-A) and side scatter area (SSC-A) to eliminate debris (left panel). Single cells were gated using forward scatter height (FSC-H) and forward scatter width (FSC-W) and side scatter height (SSC-H) and side scatter width (SSC-W): middle and right panels, respectively. (**b**) Representative flow cytometry analysis of unstained *CreERT2*$^{-/-}$ VSMCs and Mitotracker-stained VSMCs from the indicated genotypes and (**c**) mean fluorescence intensity of these cells. Data are means ± s.e.m (n=3 independent experiments). (**d**) Representative images of confocal Z stack reconstructions of *CreERT2*$^{-/-}$, *Myh11-CreERT2*$^{+/-}$, *SM-Rcan1*$^{-/-}$, and *Rcan1*$^{-/-}$ VSMCs stained with Mitotracker (scale bar: 20 µm) and (**e**) number of mitochondria per cell. Each data point denotes an individual cell, and the horizontal bars denote the mean (long bar) and the s.e.m. Data are from 4 independent experiments. (**f**) Gene expression analysis of indicated genes by RT-qPCR in aortic extracts from the indicated mice. mRNA amounts were normalized to those of *Gapdh*. Data are means ± s.e.m. (c, e, f) One-way ANOVA with Tukey multiple comparison post-hoc test except *Sdh1* mRNA quantification (Kruskal-Wallis with Dunn multiple comparison post-hoc test); *n.s.*, non-significant vs *CreERT2*$^{-/-}$.

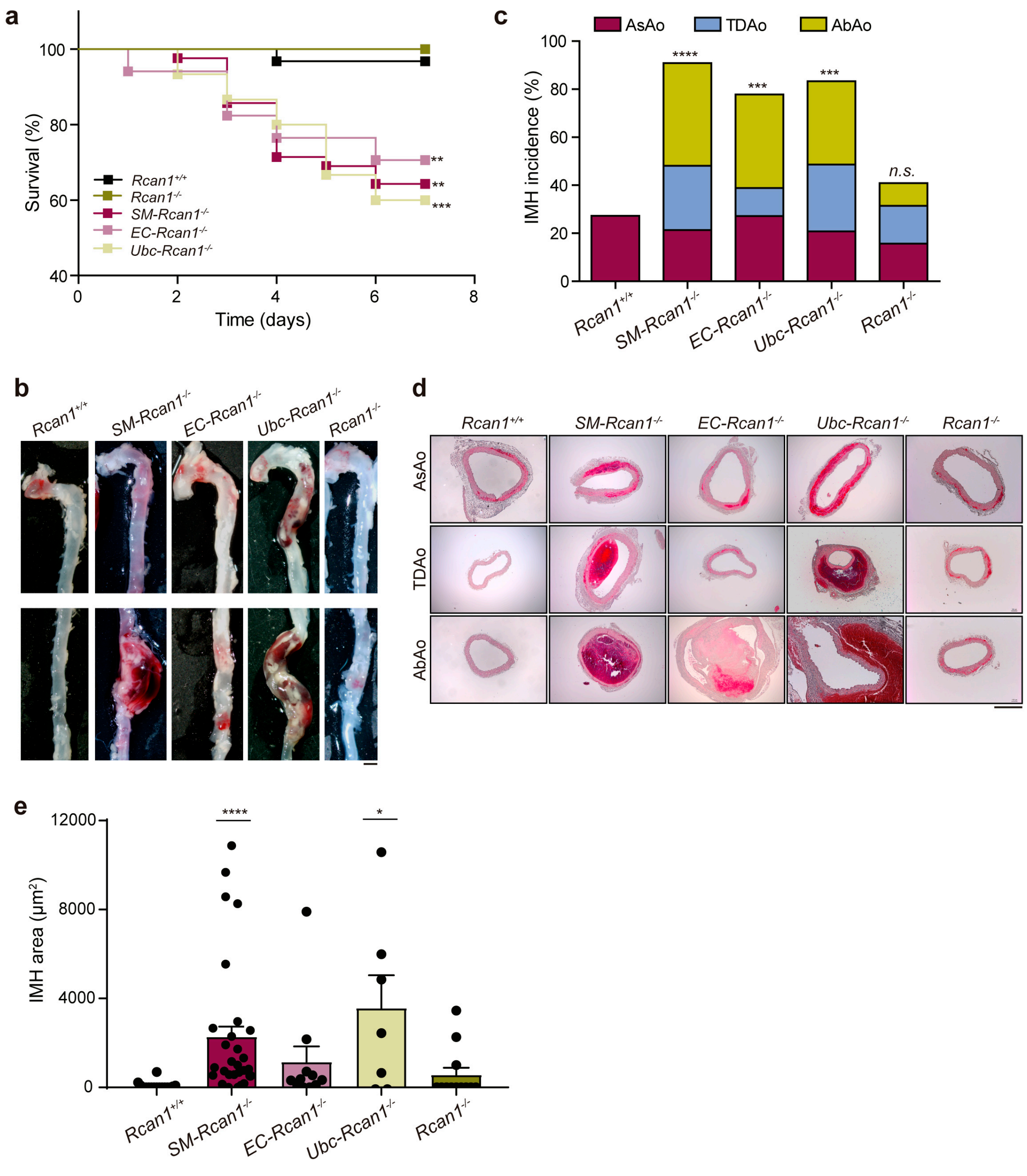

Supplementary Figure 7

Supplementary Figure 7. Aortic rupture and IMH in mice after 7 days of AngII treatment. **(a)** Survival curve of IMH group (7 days of AngII) from the long-term treatment experiment shown in Figure 3. Log-rank (Mantel-Cox) test, **p<0.01, ***p<0.001 vs *Rcan1*$^{+/+}$. All deaths were due to aortic rupture. **(b)** Representative images of macroscopic IMH. Scale bars, 1mm. **(c)** IMH incidence stratified by aortic region. Chi-square distribution, ****p<0.0001, ***p<0.001 vs *Rcan1*$^{+/+}$; *n.s.,* not significant vs *Rcan1*$^{+/+}$. **(d)** Representative images of hematoxylin-eosin stained aortic sections and **(e)** IMH area quantification in these sections. Scale bar, 500μm. *Rcan1*$^{+/+}$ (n=31), *SM-Rcan1*$^{-/-}$ (n=38), *EC-Rcan1*$^{-/-}$ (n=17), *Ubc-Rcan1*$^{-/-}$ (n=15), and *Rcan1*$^{-/-}$ mice (n=17). Each data point denotes an individual mouse, whereas histograms denote means + s.e.m. Kruskal-Wallis with Dunn multiple comparison post-hoc test, ****p<0.0001, *p<0.05 vs *Rcan1*$^{+/+}$.

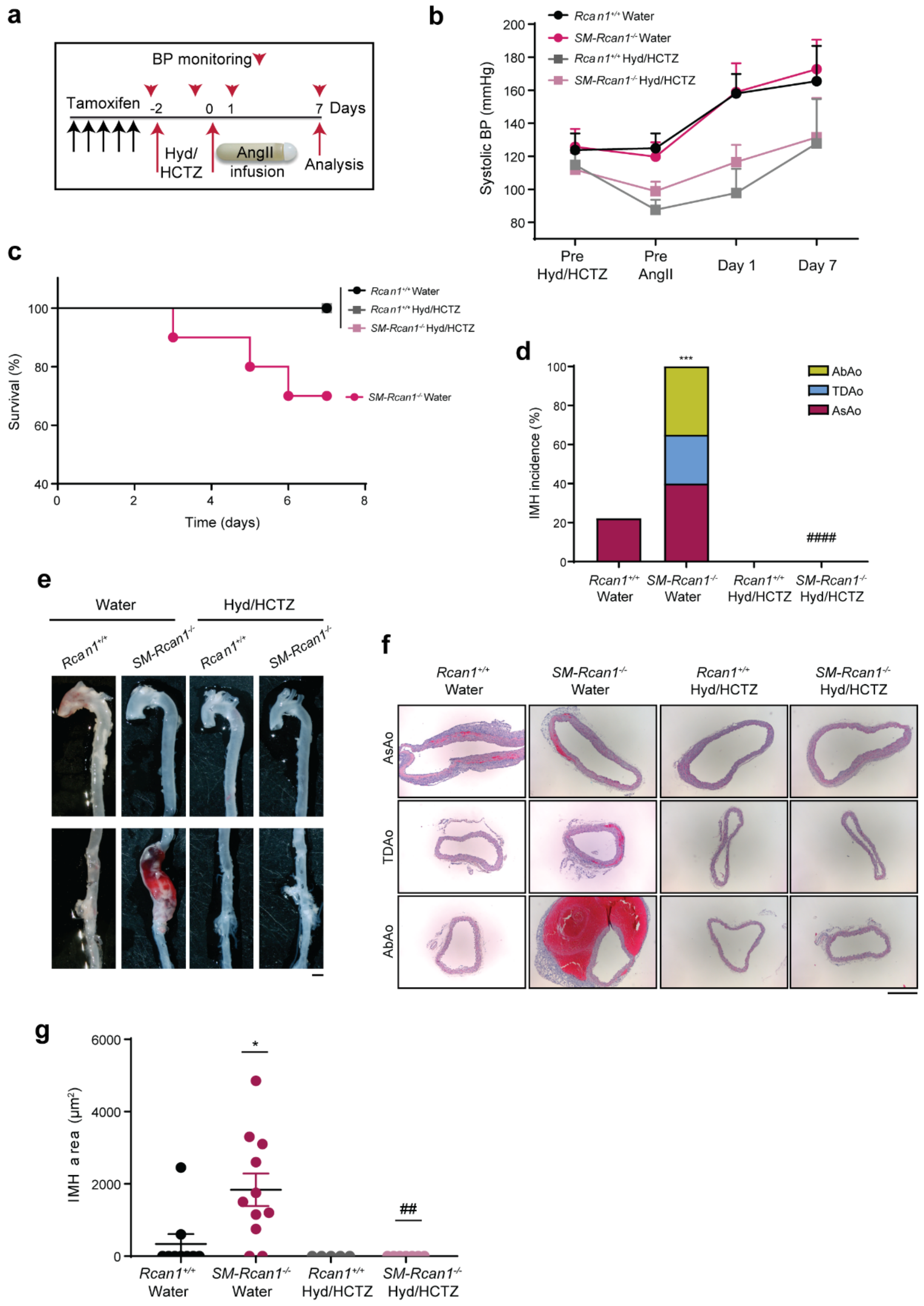

Supplementary Figure 8

Supplementary Figure 8. Vasorelaxation by hydralazine plus hydrochlorothiazide blocks AngII induction of aortic rupture and IMH in inducible *Rcan1*$^{-/-}$ mice. (a) Experimental design: 8-week-old male mice were treated with tamoxifen for 5 consecutive days and then with a vasorelaxant combination of hydralazine and hydrochlorothiazide (in the drinking water) starting 2 days before the implantation of osmotic minipumps for AngII infusion (1 µg/kg/min) and continuing for the next 7 days. **(b)** Quantification of systolic BP of *Rcan1*$^{+/+}$ mice treated with water (n=9) or the vasorelaxant combination (n=5), and *SM-Rcan1*$^{-/-}$ mice treated with water (n=10) or the vasorelaxant combination (n=7), immediately before the initiation of the vasorelaxant treatment (Pre Hyd/HCTZ) and the implantation of the osmotic minipumps (Pre Ang-II), and after 1 and 7 days of AngII treatment. Data are means ± s.e.m. **(c)** Survival curves of these mice after AngII osmotic minipump implantation. Log-rank (Mantel-Cox) test, *n.s.*, non-significant vs *CreERT2*$^{-/-}$. All deaths were due to aortic rupture. **(d)** End-of-experiment IMH incidence in the AsAo, TDAo, and AbAo. Chi-square distribution, ***p<0.001 vs *Rcan1*$^{+/+}$ Water; ####p<0.001 vs *SM-Rcan1*$^{-/-}$ Water. **(e)** Representative images of macroscopic hematomas in aortas from mice euthanized at the end of the experiment. Scale bar, 1mm. **(f)** Representative images of aortic sections from the same cohorts of mice stained with hematoxylin-eosin. Scale bar, 500µm. **(g)** Hemorrhage area in aortic sections. Each data point denotes an individual mouse, and the horizontal bars denote the mean (long bar) and the s.e.m. Kruskal-Wallis with Dunn multiple comparison post-hoc test, *p<0.05 vs *Rcan1*$^{+/+}$ Water; ##p<0.01 vs *SM-Rcan1*$^{-/-}$ Water. (c, d, g) Quantitative data for *SM-Rcan1*$^{-/-}$ Water are the same than those shown for *SM-Rcan1*$^{-/-}$ in Supplementary Figure 5, as these were the same mice and these experiments were performed at the same time.

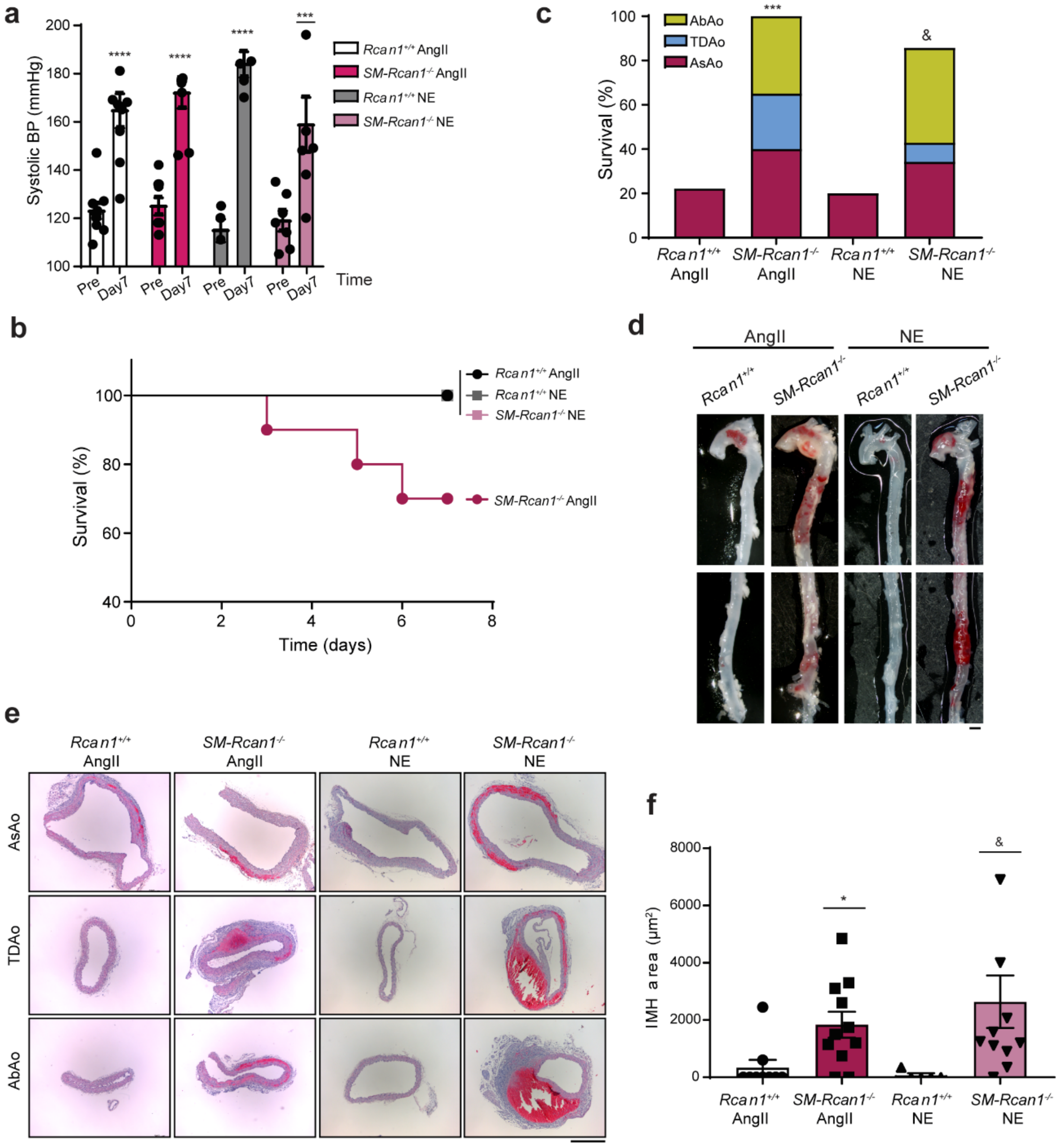

Supplementary Figure 9

Supplementary Figure 9. The norepinephrine vasopressor induces IMH in inducible *Rcan1*$^{-/-}$ mice. Eight-week-old male mice were treated with tamoxifen for 5 consecutive days before the implantation of osmotic minipumps for AngII (1 μg/kg/min) or noerpinephrine (NE) infusion (34.5mg/kg/day). **(a)** Quantification of systolic BP of *Rcan1*$^{+/+}$ and *SM-Rcan1*$^{-/-}$ mice before (Pre) and 7 days (Day 7) after treatment with AngII (n=10, each group) or NE (n=5, and n=7, respectively). Each data point denotes an individual mouse, whereas histograms denote means ± s.e.m. Two-way ANOVA with Tukey multiple comparison post-hoc test, ****p<0.0001, ***p<0.001 vs Pre for each genotype. **(b)** Survival curves of these mice after AngII or NE osmotic minipump implantation. Log-rank (Mantel-Cox) test, *n.s.*, non-significant vs *Rcan1*$^{+/+}$ Ang-II. All deaths were due to aortic rupture. **(c)** End-of-experiment IMH incidence in the AsAo, TDAo, and AbAo. Chi-square distribution, ***p<0.001 vs *Rcan1*$^{+/+}$ Ang-II: &p<0.05 vs *Rcan1*$^{+/+}$ NE. **(d)** Representative images of macroscopic hematomas in aortas from mice euthanized at the end of the experiment. Scale bar, 1mm. **(e)** Representative images of aortic sections from the same cohorts of mice stained with hematoxylin-eosin. Scale bar, 500μm. **(f)** Hemorrhage area in aortic sections. Each data point denotes an individual mouse, whereas histograms denote means ± s.e.m. Kruskal-Wallis with Dunn multiple comparison post-hoc test, *p<0.05 vs *Rcan1*$^{+/+}$ Ang-II; &p<0.05 vs *Rcan1*$^{+/+}$ NE. (b, c, f) Quantitative data for *SM-Rcan1*$^{-/-}$ Ang-II and *Rcan1*$^{+/+}$ Ang-II are the same than those shown for *SM-Rcan1*$^{-/-}$ Water and *Rcan1*$^{+/+}$ Water in Supplementary Figure 8, as these were the same mice and these experiments were performed at the same time.

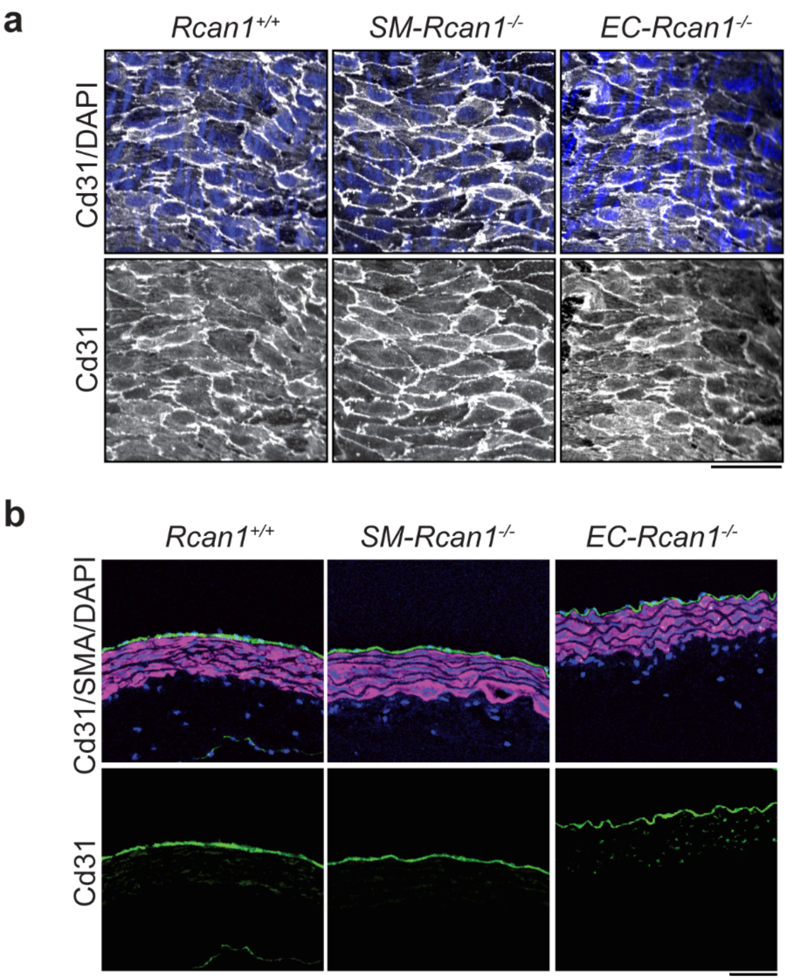

Supplementary Figure 10. Endothelial barrier integrity in conditional *Rcan1*$^{-/-}$ mice. (a) Representative images of endothelial-cell immunofluorescence staining in *en face* aortas from untreated mice (n=3 per genotype). Cd31 (gray), DAPI-stained nuclei (blue). Scale bar, 20μm. **(b)** Representative images of Cd31 (green), SMA (pink), and DAPI (blue) staining of transverse aortic sections from untreated mice (n=6 per genotype). Scale bar, 50μm.

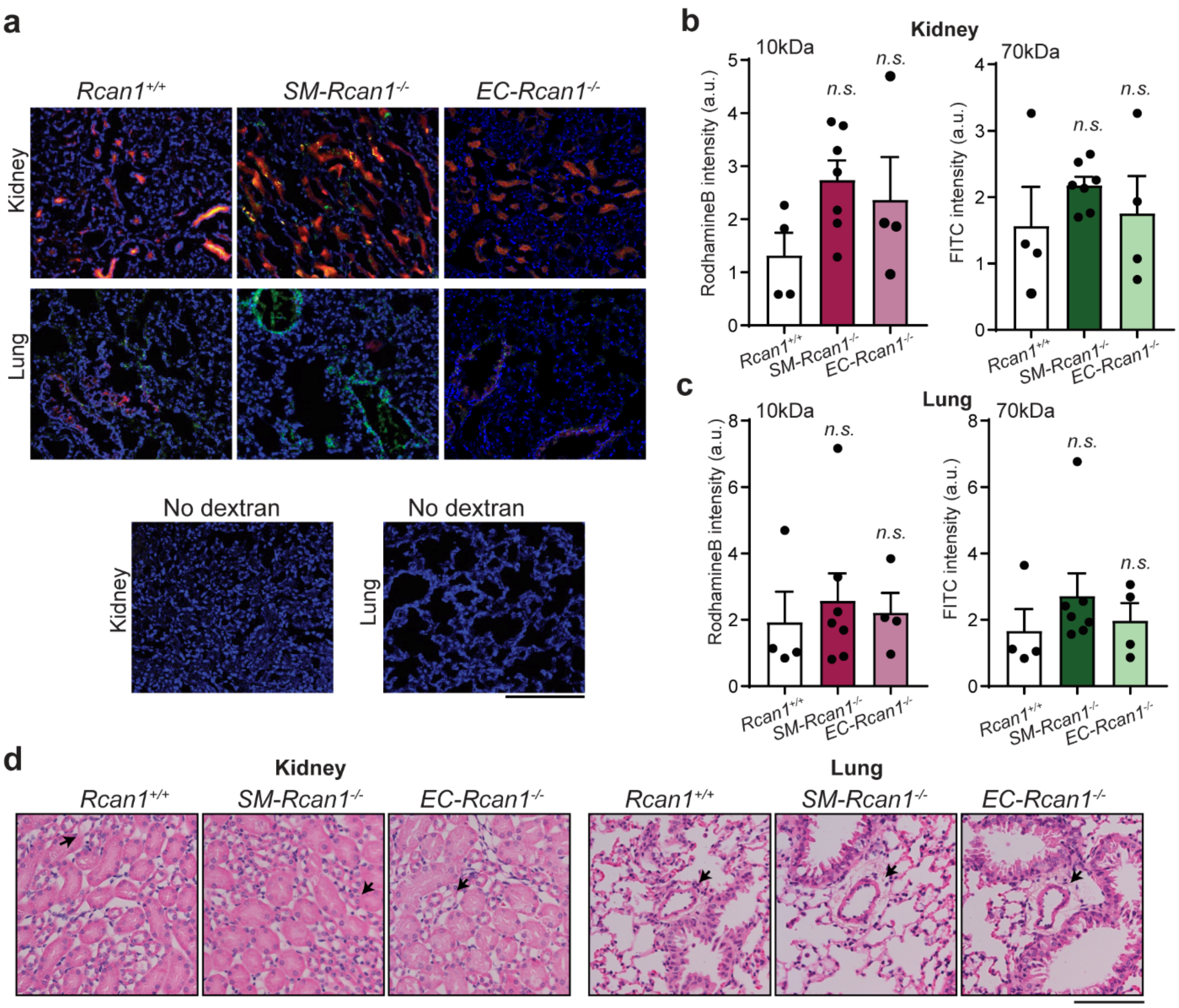

Supplementary Figure 11. Characterization of vascular permeability in kidney and lung. **(a)** Representative images of the accumulation of FITC-labeled 70-kDa dextran (green) and RhodamineB-labeled 10-kDa dextran (red) in kidney (top panels) and lung (bottom panels) sections from *Rcan1*$^{+/+}$ (n=5), *SM-Rcan1*$^{-/-}$ (n=7), and *EC-Rcan1*$^{-/-}$ mice (n=5) infused for 6h with AngII. Sections were also stained with DAPI (blue). Scale bar, 50µm. Mice injected with saline without dextran were used as a control. Fluorescence intensity quantification of labeled dextrans on **(b)** kidney and **(c)** lung sections, a.u., arbitrary units. Left, RhodamineB-labeled 10-kDa dextran measurements; right, FITC-labeled 70-kDa dextran measurements. Each data point denotes an individual mouse, whereas histograms denote means + s.e.m. One-way ANOVA with Tukey multiple comparison post-hoc test (b) Kruskal-Wallis with Dunn multiple comparison

post-hoc test (c); *n.s.*, non-significant vs *Rcan1*$^{+/+}$. *Rcan1*$^{+/+}$ (n=4), *SM-Rcan1*$^{-/-}$ (n=7), and *EC-Rcan1*$^{-/-}$ (n=4) mice. **(d)** Representative images of hematoxylin-eosin staining of sections from kidney (left) and lung (right) in *Rcan1*$^{+/+}$, *SM-Rcan1*$^{-/-}$, and *EC-Rcan1*$^{-/-}$ animals after 7 days of AngII treatment (n=4 per genotype). Scale bar, 100μm.

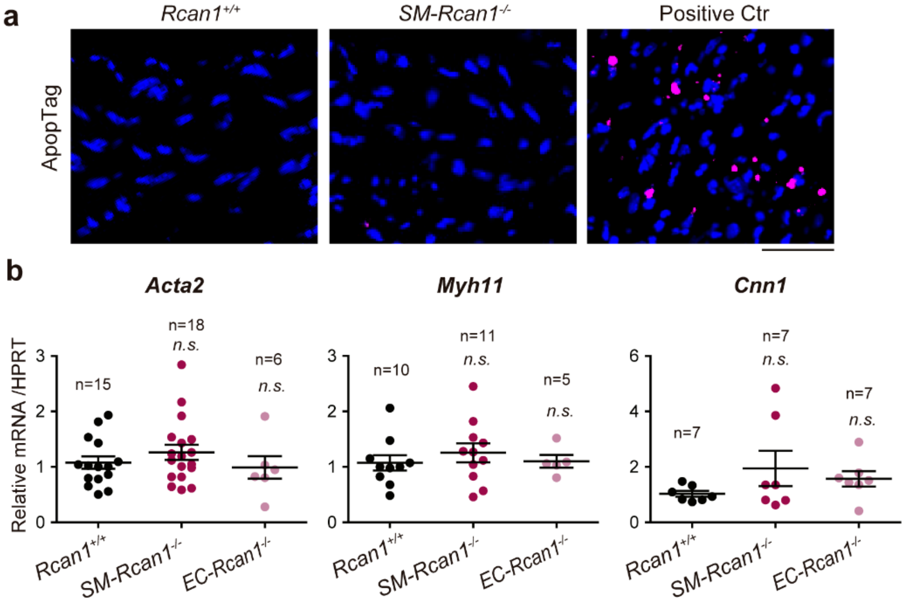

Supplementary Figure 12. Conditional *Rcan1* deletion does not induce apoptosis or contractility genes expression. (a) Representative apoptosis (pink) and DAPI (blue) staining on aortic sections from *Rcan1*$^{+/+}$ and *SM-Rcan1*$^{-/-}$ animals and from an AAA (Positive Ctr) obtained from *Apoe*$^{-/-}$ mice infused for 28 days with AngII (n=4). Scale bar, 50μm. **(b)** RT-qPCR analysis of *Acta2, Myh11*, and *Cnn1* mRNA levels in aortic extracts from the indicated mice. mRNA amounts were normalized to those of *Hprt*. Each data point denotes an individual mouse, and the horizontal bars denote the mean (long bar) and the s.e.m. Two-way ANOVA with Tukey multiple comparison post-hoc test (*Myh11*) and Kruskal-Wallis with Dunn multiple comparison post-hoc test (*Acta2* and *Cnn1*); non-significant vs *Rcan1*$^{+/+}$.

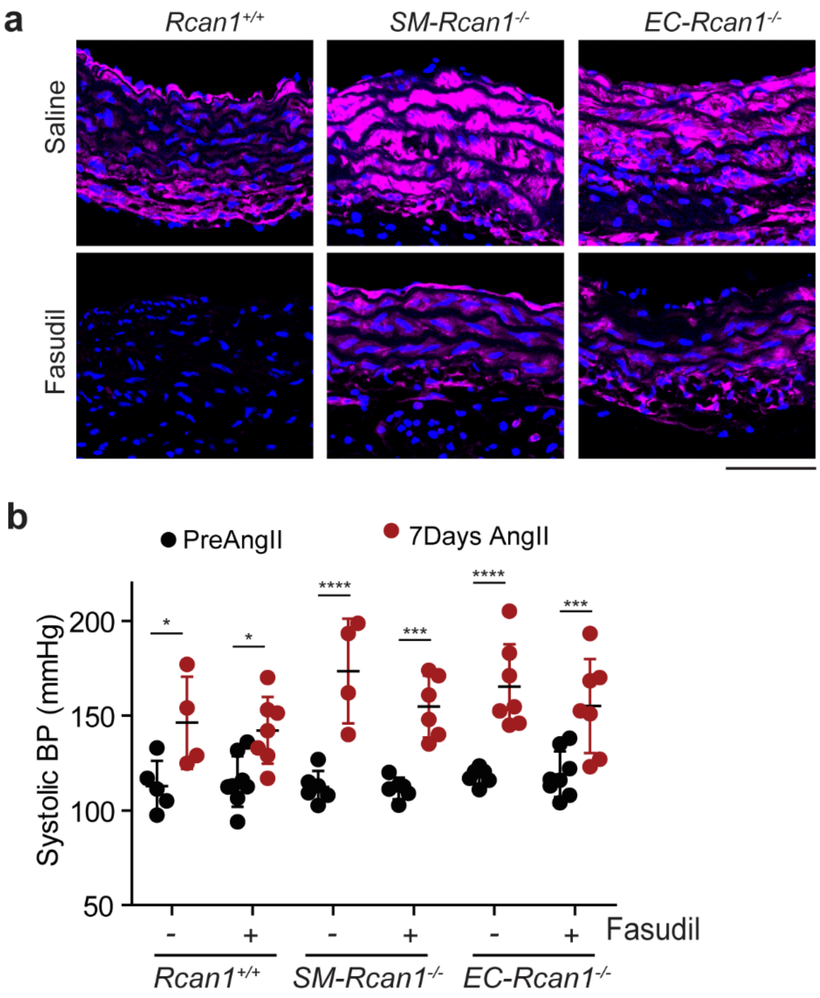

Supplementary Figure 13. Fasudil inhibits MLC activation without preventing AngII-induced hypertension. (a) Representative p-MLC immunofluorescence (pink) and DAPI (blue) staining on aortic sections from the indicated mice treated with Fasudil or saline. Scale bar, 50μm. **(b)** Systolic blood pressure (BP) in saline- (-) or Fasudil-treated (+) *Rcan1*$^{+/+}$, *SM-Rcan1*$^{-/-}$, and *EC-Rcan1*$^{-/-}$ mice before (PreAngII) and at the end of the stimulation with AngII (7Days AngII). Each data point denotes an individual mouse, and the horizontal bars denote the mean (long bar) and the s.e.m. Kruskal-Wallis with Dunn multiple comparison post-hoc test. *Rcan1*$^{+/+}$ (-, n=5), *Rcan1*$^{+/+}$ (+, n=7), *SM-Rcan1*$^{-/-}$ (-, n=6), *SM-Rcan1*$^{-/-}$ (+, n=6), *EC-Rcan1*$^{-/-}$ (-, n=7), and *EC-Rcan1*$^{-/-}$ (+, n=7).

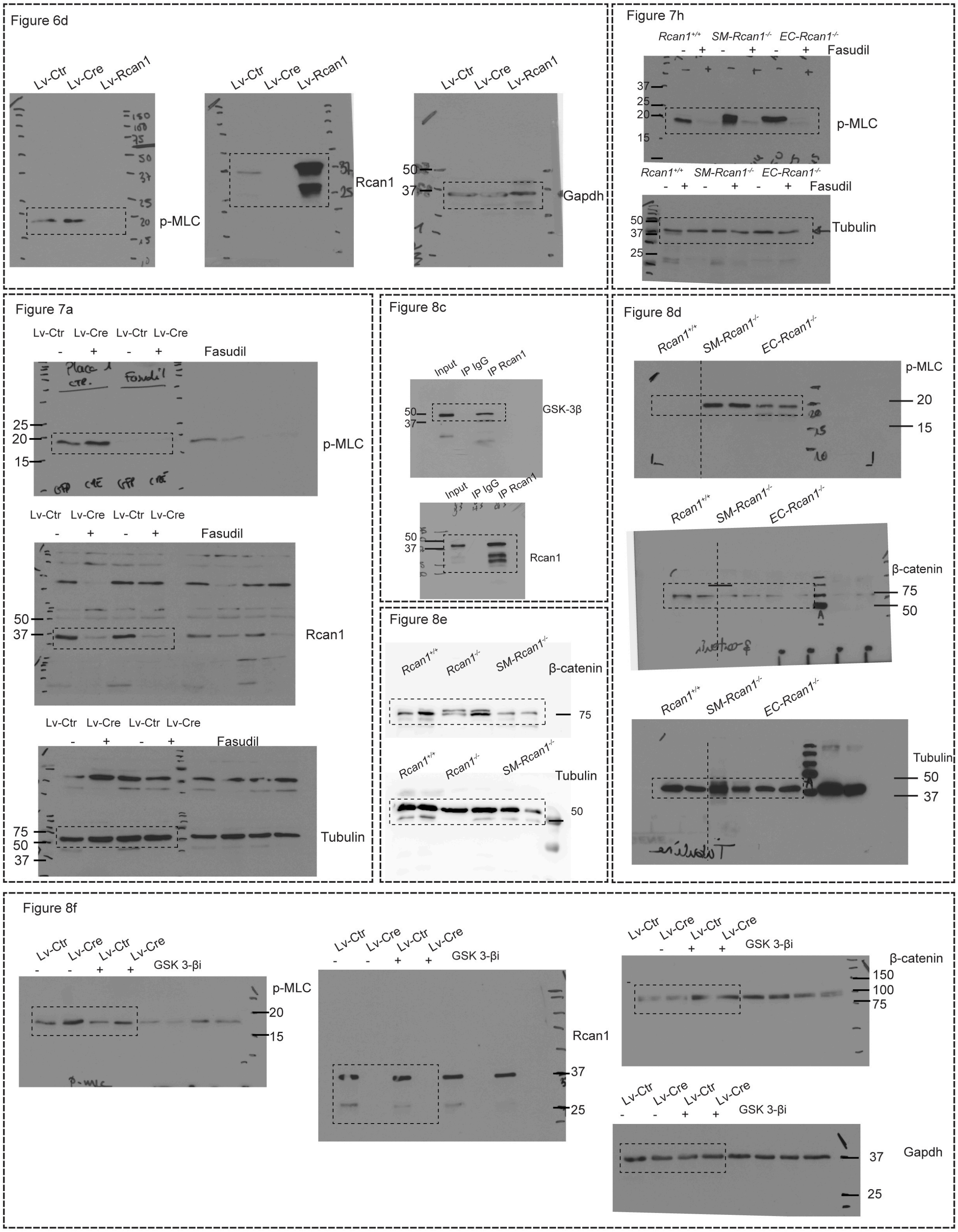

Supplementary Figure 14. Uncropped images of representative immunoblot images in Figures 6, 7 and 8.

Supplementary Figure 1f

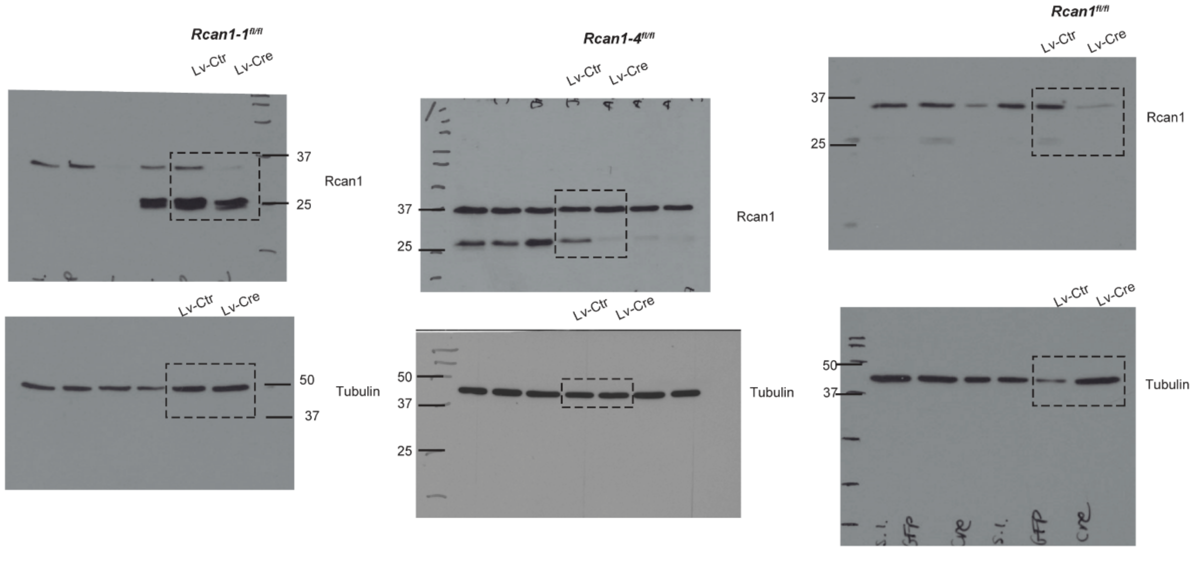

Supplementary Figure 2d

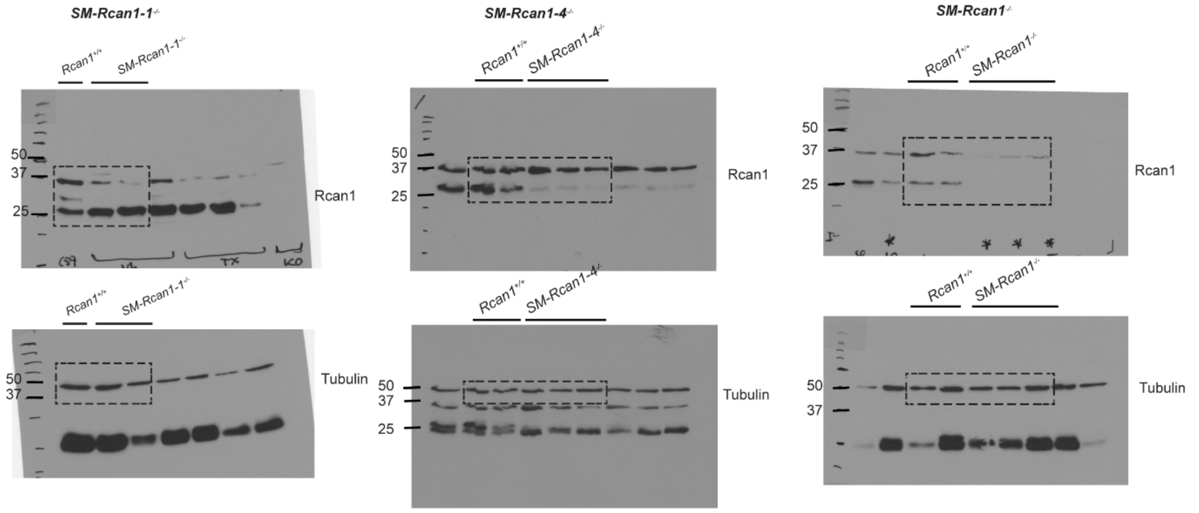

Supplementary Figure 2e

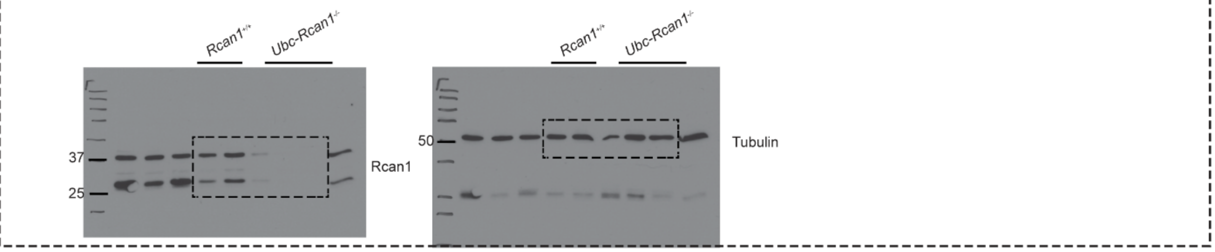

Supplementary Figure 15. Uncropped images of representative immunoblot images in Figures S1 and S2.

Locus loxP	Cre Driver	Isoform targeted	Tissue targeted	Name
Exon1	No Cre	Rcan1-1	-	*Rcan1-1* $^{fl/fl}$
Exon4	No Cre	Rcan1-4	-	*Rcan1-4* $^{fl/fl}$
Exon6	No Cre	Rcan1-1 and Rcan1-4	-	*Rcan1* $^{fl/fl}$
Exon1	*Myh11*-Cre^{ERT2}	Rcan1-1	Smooth muscle	*SM-Rcan1-1* $^{-/-}$
Exon4	*Myh11*-Cre^{ERT2}	Rcan1-4	Smooth muscle	*SM-Rcan1-4* $^{-/-}$
Exon6	*Myh11*-Cre^{ERT2}	Rcan1-1 and Rcan1-4	Smooth muscle	*SM-Rcan1* $^{-/-}$
Exon1	*Cdh5*-Cre^{ERT2}	Rcan1-1	Endothelium	*EC-Rcan1-1* $^{-/-}$
Exon4	*Cdh5*-Cre^{ERT2}	Rcan1-4	Endothelium	*EC-Rcan1-4* $^{-/-}$
Exon6	*Cdh5*-Cre^{ERT2}	Rcan1-1 and Rcan1-4	Endothelium	*EC-Rcan1* $^{-/-}$
Exon6	*Ubc*-Cre^{ERT2}	Rcan1-1 and Rcan1-4	Ubiquitous	*Ubc-Rcan1* $^{-/-}$
Exon6	No Cre	Rcan1-1 and Rcan1-4	Ubiquitous	*Rcan1* $^{-/-}$
Rosa26-LSL-YFP	*Myh11*-Cre^{ERT2}	-	Smooth muscle	Rosa26-LSL-YFP
Rosa26-LSL-Tomato	*Cdh5*-Cre^{ERT2}	-	Endothelium	Rosa26-LSL-Tomato

Supplementary Table 1. Description of transgenic mouse lines used in this study.

Primer	Sequence (5'-3')	PCR
Rcan1-1$^{fl/fl}$ Fw	CGGAGGGCGGACACTTG	Genotyping
Rcan1-1$^{fl/fl}$ Rv	GAGCGACATGCCACCATCAG	Genotyping
Rcan1-4$^{fl/fl}$ Fw	GCAAACGATGATGTCTTCAGCG	Genotyping
Rcan1-4$^{fl/fl}$ Rv	GCTCGGTATGGCCACACAC	Genotyping
Rcan1$^{fl/fl}$ Fw	GGGAGGTGTGGAGAGACTCTC	Genotyping
Rcan1$^{fl/fl}$ Rv	CTGGAGTGAGCCACACAGG	Genotyping
Rcan1$^{-/-}$ Fw	GGTGGTCCACGTGTGTGAGA	Genotyping
Rcan1$^{-/-}$ Rv	ACGTGAACAAAGGCTGGTCCT	Genotyping
Myh11-Cre^{ERT2} Fw	GCCTGCATTACCGGTCGATGCAACGA	Genotyping
Myh11-Cre$^{ERT2\ Rv}$	GTGGCAGATGGCGCGGCAACACCATT	Genotyping
Cdh5/Ubc-Cre^{ERT2} Fw	GCCTGCATTACCGGTCGATGCAACGA	Genotyping
Cdh5/Ubc-Cre$^{ERT2\ Rv}$	GTGGCAGATGGCGCGGCAACACCATT	Genotyping
Cassette Neo common	GCTGACCGCTTCCTCGTG	Genotyping
Delta Rcan1-1$^{-/-}$ Fw	GACTTGATGGCCCGAGTG	Genotyping
Delta Rcan1-1$^{-/-}$ Rv	GAGCGACATGCCACCATCAG	Genotyping
Delta Rcan1-4$^{-/-}$ Fw	CACAGCTCCTGCATTATCC	Genotyping
Delta Rcan1-4$^{-/-}$ Rv	GCTCGGTATGGCCACACAC	Genotyping
Delta Rcan1$^{-/-}$ Fw	CCATGTAACAGGTTACCACAGGC	Genotyping
Delta Rcan1$^{-/-}$ Rv	CCTGCTGGGTGAAGGGATAG	Genotyping
Myh11 Fw	GCCAATATTGAAACATATCTTCTGG	RT-PCR
Myh11 Rv	CGGCGAGCAGGTAGTAGAAG	RT-PCR
Acta2 Fw	GTGACCACAGCTGAACGTG	RT-PCR
Acta2 Rv	CCAGGGAGGAGGAAGAGG	RT-PCR
Cnn1 Fw	AACTTCATGGATGGCCTCAAA	RT-PCR
Cnn1 Rv	ACCCGGCTGCAGCTTGT	RT-PCR
Hk2 Fw	TGATCGCCTGCTTATTCACGG	RT-PCR
Hk2 Rv	AACCGCCTAGAAATCTCCAGA	RT-PCR
Atp5b Fw	GGTTCATCCTGCCAGAGACTA	RT-PCR
Atp5b Rv	AATCCCTCATCGAACTGGACG	RT-PCR
Sdh1 Fw	CTTGAATCCCTGCTCTGTGG	RT-PCR
Sdh1 Rv	AAAGCTGAGAGTGCCAAGAG	RT-PCR
Mtco1 Fw	CTCGCCTAATTTATTCCACTTCA	RT-PCR
Mtco1 Rv	GGGGCTAGGGGTAGGGTTAT	RT-PCR
Gapdh Fw	TGCACCACCAACTGCTTAGC	RT-PCR
Gapdh Rv	GGCATGGACTGTGGTCATGAG	RT-PCR
Hprt Fw	GCTGGTGAAAAGGACCTCT	RT-PCR
Hprt Rv	CACAGGACTAGAACACCTGC	RT-PCR

Supplementary Table 2. Name and sequence of primers used for genotyping and RT-PCR.
